# CALR promotes corneal epithelial cell proliferation and migration through Wnt7a

**DOI:** 10.1007/s11033-025-10810-x

**Published:** 2025-07-16

**Authors:** Qiaoling Wang, Qian Li, Ning Wei

**Affiliations:** Department of Ophthalmology, The Second People’s Hospital of Jinan, No.148, Jingyi Road, Jinan, 250000 Shandong Province China

**Keywords:** Corneal epithelial wound healing, CALR, Proliferation, Migration, Wnt7a

## Abstract

**Objective:**

The repair of corneal epithelial injury is essential to maintain the cornea integrity and transparency, and the molecular regulation mechanism is still unclear. CALR promotes wound healing through a variety of biological effects. Therefore, this study explored effect and mechanism of CALR on corneal epithelial wound healing.

**Methods:**

The model of repairing corneal epithelium injury in mice was established, and corneal epithelial tissues were collected from the model group and the control group. oe-CALR or sh-Wnt7a was transfected into HCE-2[50.B1] cells by Lipofectamine 2000 to over-express CALR or knock down Wnt7a in vitro. CALR mRNA expression was detected by RT-qPCR. CCK-8, clone formation assay, cell senescence, flow cytometry, wound healing and Transwell migration assays were used to detect the changes in proliferation, cell senescence, cell cycle and cell migration after transfection. CALR, Wnt7a and β-catenin proteins expression were detected by Western blot. Interaction between CALR and Wnt7a was detected by Co-immunoprecipitation.

**Results:**

CALR expression was increased in mice corneal epithelial injury repair, suggesting that CALR might play vital role in this process. CALR overexpression promoted HCE-2[50.B1] proliferation and migration, inhibited cell senescence of HCE-2[50.B1], and relieved S phase block and increased the number of HCE-2[50] cells in G0/G1 phase. Wnt7a and CALR proteins expression were respectively detected in the protein complexes co-precipitated by anti-CALR antibody and anti-Flag antibody. The interaction between CALR and Wnt7a could activate the downstream β-catenin signaling pathway. Wnt7a knockdown attenuated the effect of CALR overexpression on HCE-2[50.B1] cells proliferation, senescence and migration.

**Conclusion:**

CALR promotes proliferation and migration, inhibited senescence of HCE-2[50.B1] cells by Wnt7a, thus promoting corneal epithelial wound healing. This study will provide a theoretical basis for mechanism of CALR in corneal injury repair, and provide a new target for corneal injury clinical treatment.

**Supplementary Information:**

The online version contains supplementary material available at 10.1007/s11033-025-10810-x.

## Introduction

The cornea is an important part of eye refractive imaging, located in the front of the eye, and often exposed, so it is prone to external damage [1]. The cornea has no blood vessels, and the repair process of the cornea is slower than other tissues, so it is important that the wound heal as quickly as possible to stop the disease from progressing further [2, 3]. As outermost layer of cornea, epithelium plays vital role in the immunity, protection and structural stability of eye [4]. After the corneal epithelium is damaged, it is easy to develop infection [4]. Corneal epithelial injury occurs frequently and serious injury is difficult to treat, which often leads to keratopathy and visual impairment [5]. The major manifestations of severe corneal epithelial injury are necrosis and exfoliation of corneal epithelium, stromal edema, destruction of inner layer and conversion of incomplete dead endothelial cells into fibroblasts, which lead to the proliferation of neovascularization [2]. Its treatment methods include surgical limbal epithelial allograft and drugs to suppress inflammation and maintain corneal integrity after reconstruction [6]. Corneal injury repair is a very complex process, and its complete mechanism and potential gene control aren’t fully elucidated. At present, the mechanism of limbal stem cells in corneal injury repair is relatively in-depth, but the mechanism of corneal epithelial cells in corneal injury repair is relatively scarce.

Calreticulin (CALR) is a highly evolutionarily conserved calcium-binding protein present in the endoplasmic reticulum and relates to many cells physiological and pathological processes [7]. CALR is an endoplasmic reticulum chaperone protein involved in regulating intracellular calcium ion homeostasis and cell adhesion, which can maintain intracellular calcium ion homeostasis and assist protein folding, and plays an important role in immune regulation, cell phagocytosis, cell proliferation and apoptosis, and fibrosis [8]. More and more studies have found that CALR expression relates to tumor cells proliferation and metastasis [9, 10]. In addition, CALR is involved in wound healing [8, 11, 12]. And CALR has been shown to promote corneal wound healing and reduce fibrosis [13]. However, there are relatively few studies on CALR as a therapeutic target, and its application in clinical trials has not been reported. Therefore, studying the specific role and mechanism of CALR is of great importance for exploring therapeutic strategies targeting CALR, which is conducive to further exploring the role of CALR in wound healing.

Wnt protein is a kind of secreted glycoprotein, which is involved in cell proliferation, differentiation, polarity, motility and migration, and involves in embryonic development and maintenance of human tissue homeostasis [14]. Wnt family plays vital role in tumor, muscle regeneration, nervous system repair, reproductive system, trunk and dorsal limb formation and hind limb development, cartilage and cornea repair, and heart development [15]. Wnt7a is an important member of the Wnt family [16]. Wnt7a plays an important role in nerve development, axon and synaptic growth [17]. In wound corneas, Wnt7a expression can be rapidly induced and initiates the proliferation of corneal epithelial cells, thus enhancing wound healing [18]. However, potential mechanism of Wnt7a in corneal wound healing isn’t fully understood.

This study predicted interaction between CALR and Wnt7a through HitPredict database. Therefore, this study aims to investigate whether CALR affects corneal epithelial wound healing by regulating Wnt7a, and further explore the mechanism of CALR in corneal epithelial wound, thus providing experimental data for laboratory research and clinical treatment of corneal epithelial wound.

## Materials and methods

### Construction of mouse corneal epithelial injury model

Five 8-week-old male C57BL/6J wild-type mice were purchased from the Laboratory Animal Department of China Medical University. This study was approved by the Animal Ethics Committee of the Second People’s Hospital of Jinan (Approval number: JNEYE20230625). Experiments were conducted in compliance with National Institutes of Health guidelines and followed procedures approved by the Second People’s Hospital of Jinan Animal Care and Use Committee. All studies reported herein were performed in accordance with ARRIVE guidelines (https://arriveguidelines.org). A compound solution of ketamine and toluenthiazide was used as anesthetic, and mice were given general anesthesia by intraperitoneal injection. The eyeballs were fully exposed, the eyes were anesthetized with promecaine hydrochloride eye drops. On the right eye, Algerbrush is used to scrape off the corneal epithelium along the limbus towards the center of the cornea and apply erythromycin eye cream to reduce the risk of infection. The left eye was the control group without mechanical injury. About 48 h later, the corneal injury area healed. The corneal epithelium of both eyes was scraped, the healing tissue of the right eye was used as the model group, and the corneal epithelium of the left eye was used as the control group.

### Cell culture and transfection

Human corneal epithelial cell line HCE-2[50.B1] (CRL-11135), purchased from the ATCC (VA, USA). The cells were cultured in DMEM/F12 medium (Gibco, USA) containing 10% FBS (Gibco, USA) and 100 U·mL^−1^ penicillin–streptomycin (Solarbio, China), supplemented with 5 ng/mL of epidermal growth factor (EGF) (Gibco, USA) and 5 μg/mL insulin in a 37℃, 5% CO_2_ incubator.

CALR overexpression (oe-CALR), short hairpin RNA of Wnt7a (sh-Wnt7a), Wnt7a overexpression (pc-Wnt7a-Flag) and its corresponding control (oe-NC, sh-NC and pc-NC) vectors were obtained from GenePharma (Shanghai, China). After 24 h, these vectors were separately or co-transfected into HCE-2[50.B1] cells by Lipofectamine 2000 (Invitrogen, USA). After 48 h of transfection, drug screening was performed in a culture medium containing corresponding antibiotics. After screening, the obtained cells were expanded and cultured for subsequent experiments.

### RT-qPCR

Total RNA of tissue specimens and cells was extracted by the Trizol method. The procedure of reverse transcription  was performed strictly according to the instructions of cDNA reverse transcriptase kit (Takara, Japan). RNA was reverse transcribed into cDNA, which was used as a template for amplification. The relative mRNA expression was detected by SYBR Premix Ex Taq II detection kit (Takara, Japan) in a model 7300 real-time PCR instrument (ABI, USA). Relative gene expression was calculated by 2^−ΔΔCt^ method with GAPDH as an internal control.

### Western blot

Total protein was extracted by lysis of tissues and treated cells using RIPA buffer (Solarbio, China). The nuclear and cytoplasmic proteins were separated using the kit (NE-PER Nuclear and Cytoplasmic Extraction Reagents, Thermo, USA). The BCA kit (Beyotime, China) detected the extracted proteins concentration. Extracted proteins were mixed thoroughly with 5 × Loading buffer and boiled for 5 min. Equal amounts of protein samples were then separated by electrophoresis on a 10% SDS-PAGE and transferred to PVDF membrane (Bio-Rad, USA). After blocking with 5% skim milk for 1 h at room temperature, PVDF membranes were incubated with primary antibodies (CALR (Proteintech, USA), Wnt7a (Proteintech, USA), Flag (Proteintech, USA), β-catenin (Abcam, USA), PCNA (Abcam, USA) and GAPDH (Abcam, USA), 1: 1000) overnight at 4 °C. Subsequently, corresponding secondary antibodies (Abcam, USA) were added and incubated at room temperature for 1 h. Protein bands were visualized by enhanced chemiluminescence.

### Co-immunoprecipitation

Protein samples were extracted with IP lysis/wash buffer using the Pierce™ Classical Magnetic Bead Method IP/Co-IP Kit (Thermo Scientific, USA), and cell lysates were incubated with antibodies at 4 °C overnight. Antigen–antibody complexes were allowed to bind to protein A/G magnetic beads for 1 h at room temperature. Protein samples eluted from the eluate were then boiled after adding loading buffer, and the samples were examined by western blot.

### CCK-8 assay

The experiment was performed when cells were seeded with 1 × 10^4^ cells/well in 96-well plates and density was 80% and evenly distributed. The old medium was discarded, washed gently with PBS, and replaced with new medium. After 24, 48, and 72 h culture, 10 μl of CCK-8 reagent (Dojindo, China) was added and continued to be cultured for 2 h. Cell viability was then calculated by measuring OD value at 450 nm using a microplate reader.

### Clone formation assay

Cells were seeded in 6-well plates (2 × 10^3^ cells/well) and cultured for 2–3 weeks until macroscopic monoclonal clusters of cells were grown. The culture was stopped when monoclonal clusters of cells could be counted visually. Cells were fixed with 4% paraformaldehyde and stained with crystal violet for 15 min, rinsed with running water, and dried. The results of the experiments on six-well plates were photographed by stereoscopic microscope. The clone clusters on the photos were counted by Image J and the data differences between groups were analyzed.

### Cell senescence assay

Cell senescence rate was detected by β-galactosidase (β-GAL) kit (Beyotime, China). Cells were seeded with 3 × 10^5^ cells/well in 6-well plates. After washing once with PBS, fixative solution was added and fixed for 15 min at room temperature. β-GAL staining solution was added, and 6-well plates were sealed with a sealing membrane and incubated overnight at 37 °C, CO_2_ free incubator in dark. The positive cells were observed and photographed by inverted fluorescence phase contrast microscope. Five fields were randomly selected from each group of samples, and positive cells percentage was calculated.

### Cell cycle was detected by flow cytometry

After transfection, cells were harvested by 0.25% trypsin digestion and fixed overnight with 70% ethanol. The supernatant was discarded by centrifugation, washed once with PBS, and centrifuged again. Cells were stained with a PI staining kit (Beyotime, China). Cells percentage in different cycles was determined by flow cytometry.

### Wound healing assay

Cells were seeded with 1 × 10^6^ cells/well of a 6-well cell plate. The next day, whether the cells were fused or not was observed. If the fusion rate reached 100%, the cells within the equal width distance of each well were scraped with a 100 μl gun tip, and the scratch width was recorded by photographing. After 24 h of cultivation, scratch width was recorded again. The photographs of the cells were processed and analyzed using Image J software.

### Transwell migration assay

Concentration of cells was adjusted to 3 × 10^5^cells/ml with serum-free DMEM/F12 medium. A 24-well cell culture plate was taken, and 500 μl DMEM/F12 complete culture medium was added into the well. A transwell chamber (Corning, USA) was placed into the well, and there were no bubbles between the chamber and the liquid surface of the culture medium. 100 μl of cell suspension was added to the chamber and incubated for 24 h. Cells were fixed with 4% paraformaldehyde for 30 min and stained with crystal violet for 30 min. The upper chamber cells were carefully rubbed using a cotton swab. The cells that pass through the membrane were counted.

### Statistical analysis

All data were processed by GraphPad Prism 6.0 software, and data processing was expressed as mean ± standard deviation (mean ± SD). T test was used for comparison between groups, and *P* < 0.05 was considered statistically significant.

## Results

### Expression of CALR in the process of corneal epithelial injury repair

**Fig. 1:**
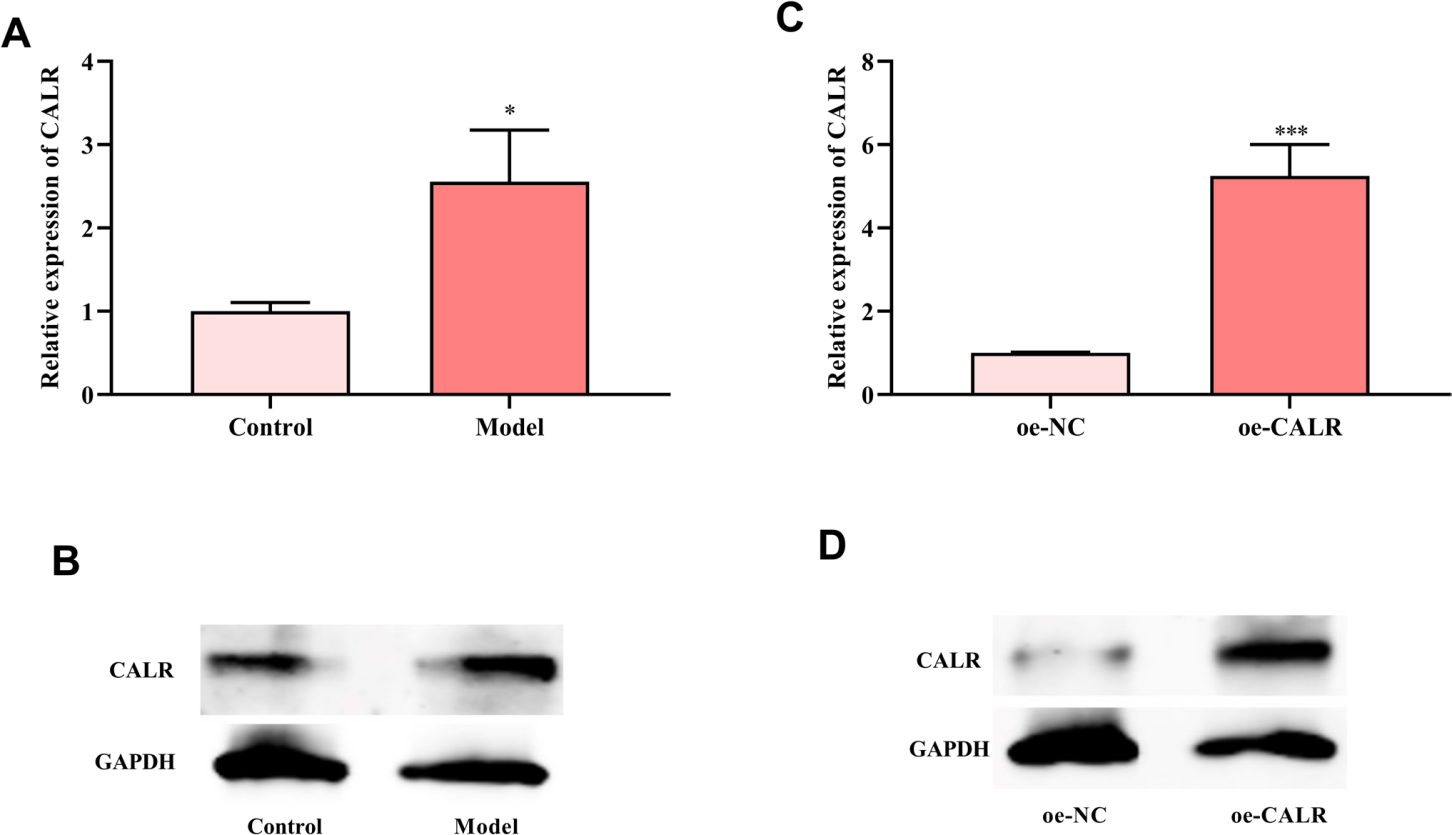
CALR expression in corneal epithelial injury repair mice model and HCE-2[50.B1] cells. **A**, **B** CALR mRNA and protein expressions in corneal epithelial injury repair mice model. **C**, **D** CALR expression in HCE-2[50.B1] cells after oe-CALR or oe-NC transfection. *P < 0.05, ***P < 0.001, compared with control or oe-NC group

48 h after corneal epithelial injury repair model was successfully established, the corneal epithelial tissues of the injured eyes and the control eyes were collected. CALR expression in the model group was increased versus control group (Fig. 1A-B).

To investigate whether CALR can promote corneal epithelial repair of corneal injury and what is the reason why CALR promotes corneal injury repair, we first examined the effect of CALR on corneal epithelial cells by in vitro cell experiments. HCE-2[50.B1] cells were used to construct the human corneal epithelial cell line overexpressing CALR (oe-CALR). RT-qPCR and western blot verified that the transfection was successful (Fig. 1C-D).

### Effect of CALR on the proliferation ability and senescent of HCE-2[50.B1] cells

**Fig. 2:**
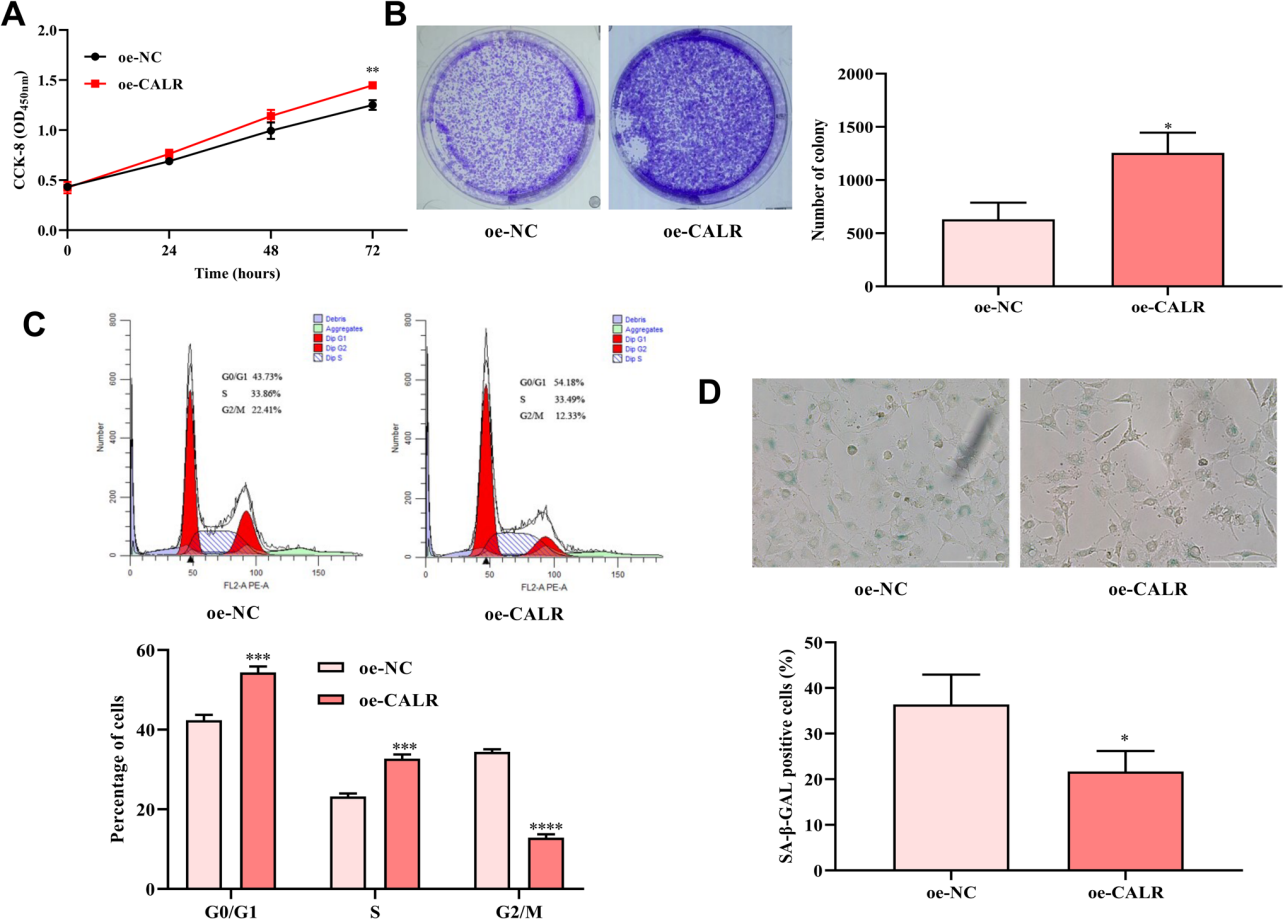
Effect of CALR overexpression on proliferation and senescence of HCE-2[50.B1] cells. **A**, **B** CCK-8 and colony formation assays evaluated the effect of CALR overexpression on HCE-2[50.B1] cells proliferation. **C** Effect of CALR overexpression on HCE-2[50.B1] cells cycle. **D** Effect of CALR overexpression on HCE-2[50.B1] cells senescence. *P < 0.05, **P < 0.01, ***P < 0.001, ****P < 0.0001, compared with oe-NC group

Compared with oe-NC group, the OD_450_ values of oe-CALR group were increased after 24, 48, and 72 h of culture (Fig. 2A). Cell clones number in oe-CALR group was increased versus oe-NC group (Fig. 2B). Proportion of cells in S and G0/G1 phase in oe-CALR group were increased versus oe-NC group (Fig. 2C). Moreover, proportion of senescent cells in oe-CALR group was notably less than that of oe-NC group (Fig. 2D).

### Effect of CALR on the migration ability of HCE-2[50.B1] cells

**Fig. 3:**
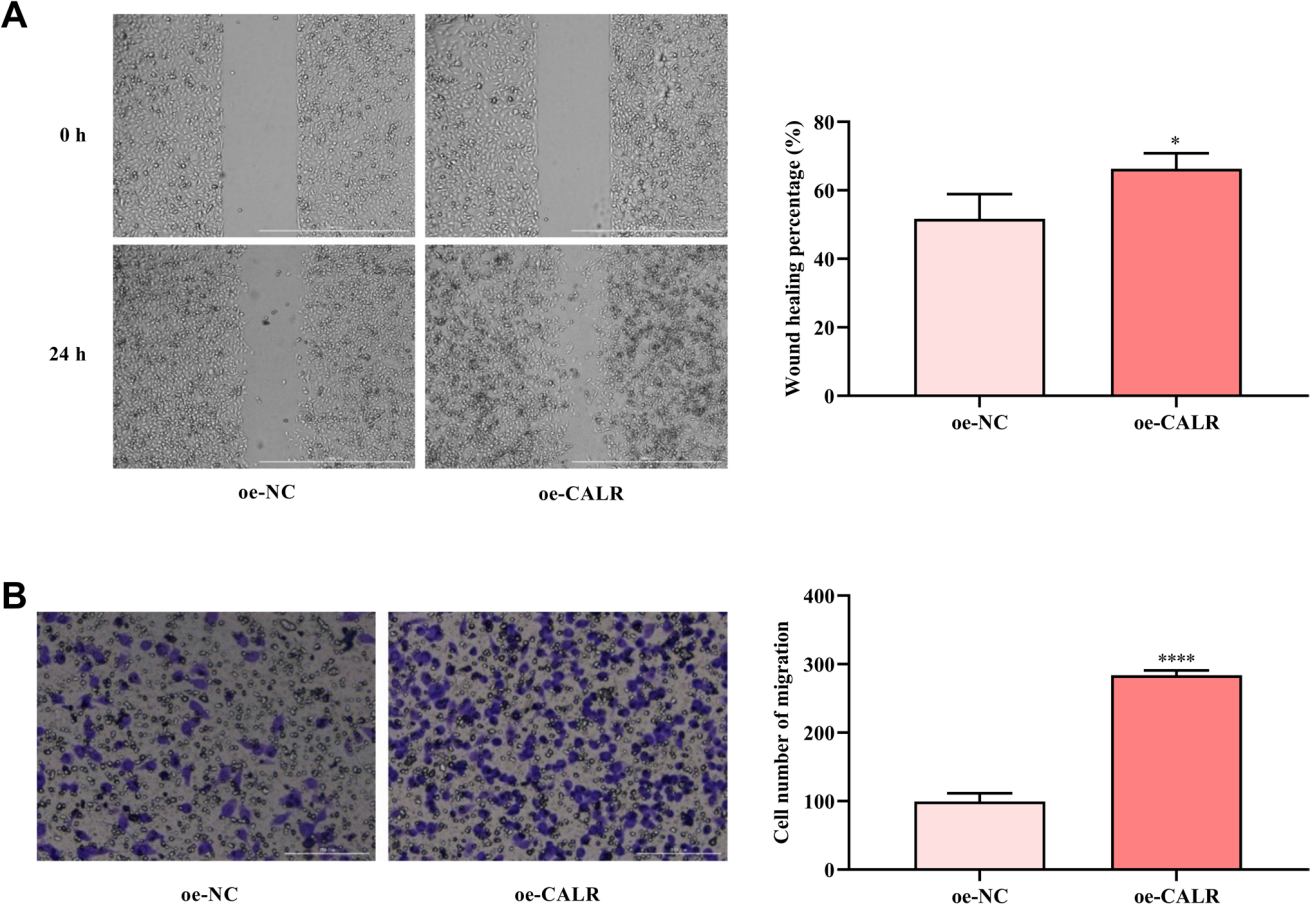
Effect of CALR overexpression on migration of HCE-2[50.B1] cells. **A**, **B** Wound healing and Transwell assays was used to detect the effect of CALR overexpression on the migration of HCE-2[50.B1] cells. *P < 0.05, ****P < 0.0001, compared with oe-NC group

The migration ability of corneal epithelial cells is an important part of the wound healing process. In this study, whether CALR affects migration ability of HCE-2[50.B1] cells was detected by wound healing and Transwell migration in vitro. The migration distance rate of HCE-2[50.B1] cells was increased after CALR overexpression at the same healing time (Fig. 3A). Transwell chamber migration results showed that the number of transmembrane HCE-2[50.B1] cells increased after transfection of oe-CALR (Fig. 3B). These results indicated that the migration ability of HCE-2[50.B1] cells was promoted after transfection of oe-CALR.

### CALR interacts with Wnt7a

**Fig. 4:**
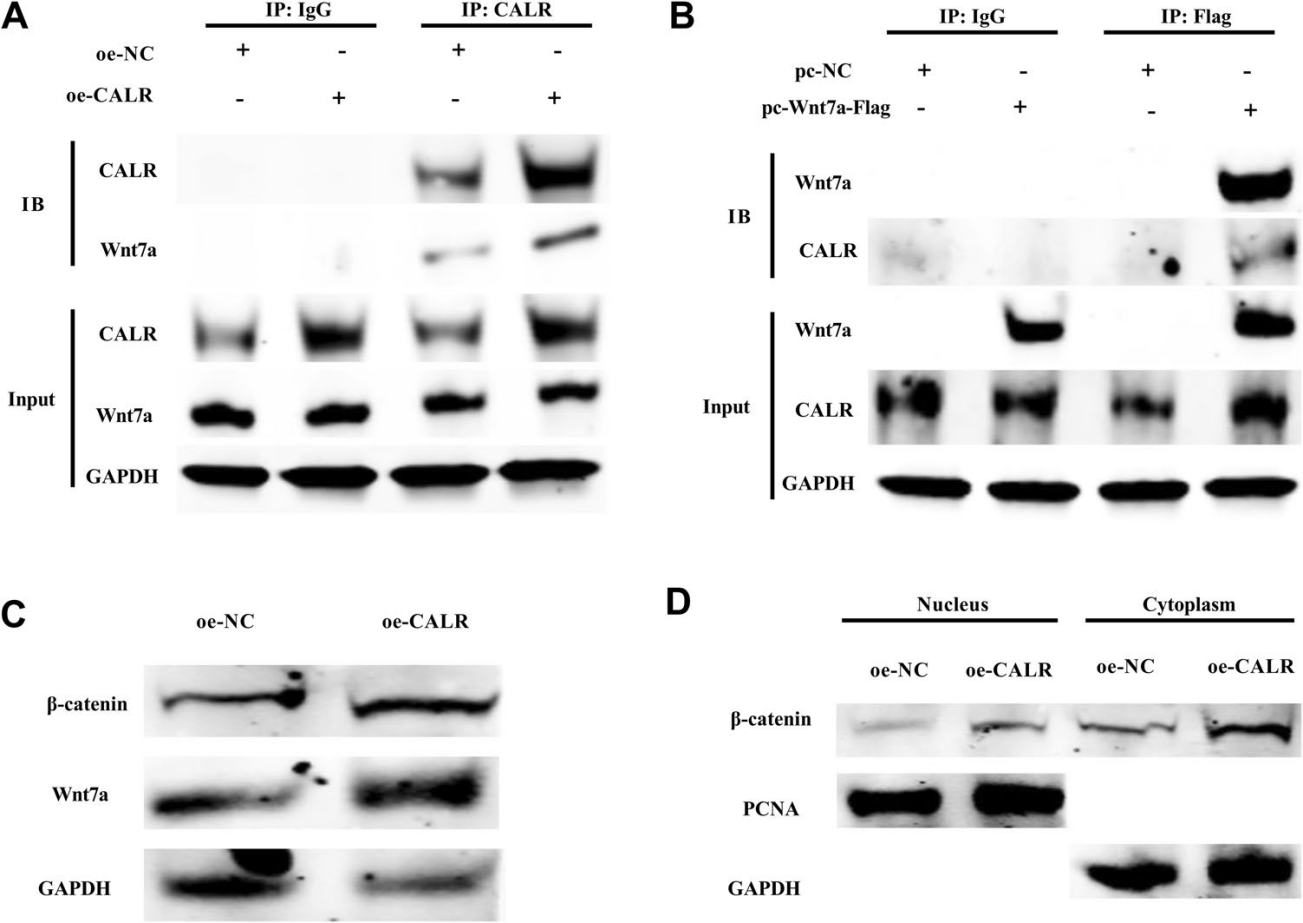
CALR can interact with Wnt7a. **A**, **B** Co-immunoprecipitation of CALR and Wnt7a in HCE-2[50.B1] cells was analyzed by western blot. **C** Effect of CALR overexpression on β-catenin and Wnt7a expression in HCE-2[50.B1] cells. **D** Effect of CALR overexpression on β-catenin expression in the nucleus and cytoplasm of HCE-2[50.B1] cells

CALR overexpression promoted the proliferation and migration of HCE-2[50.B1] cells. To clarify the biological pathways mediating the CALR function in promoting HCE-2[50.B1] cells proliferation and migration, the interaction protein network of CALR was obtained by HitPredict database. Wnt7a was found to be a potential interacting protein of CALR. Results of co-immunoprecipitation showed that the protein bands of Wnt7a could be detected in the co-precipitation complex captured by anti-CALR antibody (Fig. 4A). Similarly, CALR protein bands were detectable after protein co-precipitation complexes from cell lysis were captured with anti-Flag antibody (Fig. 4B). This suggests that CALR and Wnt7a could interact in HCE-2[50.B1] cells. The effect of CALR overexpression on Wnt7a expression and its influence on the downstream β-catenin expression of Wnt were also explored. CALR overexpression increased the secretion of wnt7a, thereby activating the expression of β-catenin downstream of the Wnt pathway (Fig. 4C). Moreover, CALR overexpression not only increased the accumulation of β-catenin in the cytoplasm but also activated the nuclear entry of β-catenin (Fig. 4D). The interaction between CALR and Wnt7a could activate the downstream β-catenin signaling pathway.

### Wnt7a knockdown reversed the affection of CALR overexpression on HCE-2[50.B1] cells proliferation and senescence

**Fig. 5:**
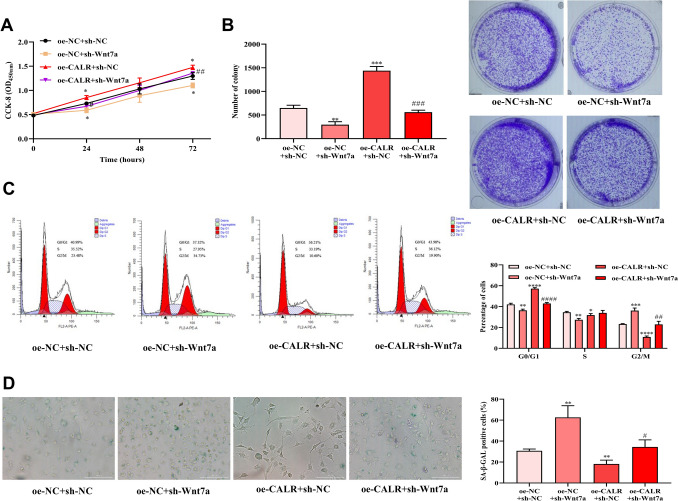
Wnt7a knockdown reversed the effects of CALR overexpression on the proliferation and senescence of HCE-2[50.B1] cells. **A**, **B** Effect of oe-CALR and sh-Wnt7a co-transfection on the proliferation of HCE-2[50.B1] cells. **C**, **D** After Wnt7a knockdown in HCE-2[50.B1] cells with oe-CALR, the changes of cells cycle and senescence were detected. *P < 0.05, **P < 0.01, ***P < 0.001, ****P < 0.0001, compared with oe-NC + sh-NC group. #*P* < 0.05, ##*P* < 0.01, ###*P* < 0.001, ####*P* < 0.0001, compared with oe-CALR + sh-NC group

To further investigate whether CALR promotes corneal epithelial cells proliferation through Wnt7a, Wnt7a downregulation by transfection of sh-Wnt7a was performed in HCE-2[50.B1] cells with CALR overexpression. Compared with the control group, CALR overexpression and Wnt7a inhibition reduced the proliferation ability, colony formation ability, and increased the S and G2/M phase cells proportion and cell senescence (Fig. 5). These results suggested that CALR promoted HCE-2[50.B1] cells proliferation and inhibited senescence through Wnt7a.

### Effect of CALR overexpression on HCE-2[50.B1] cells migration was attenuated by Wnt7a knockdown

**Fig. 6:**
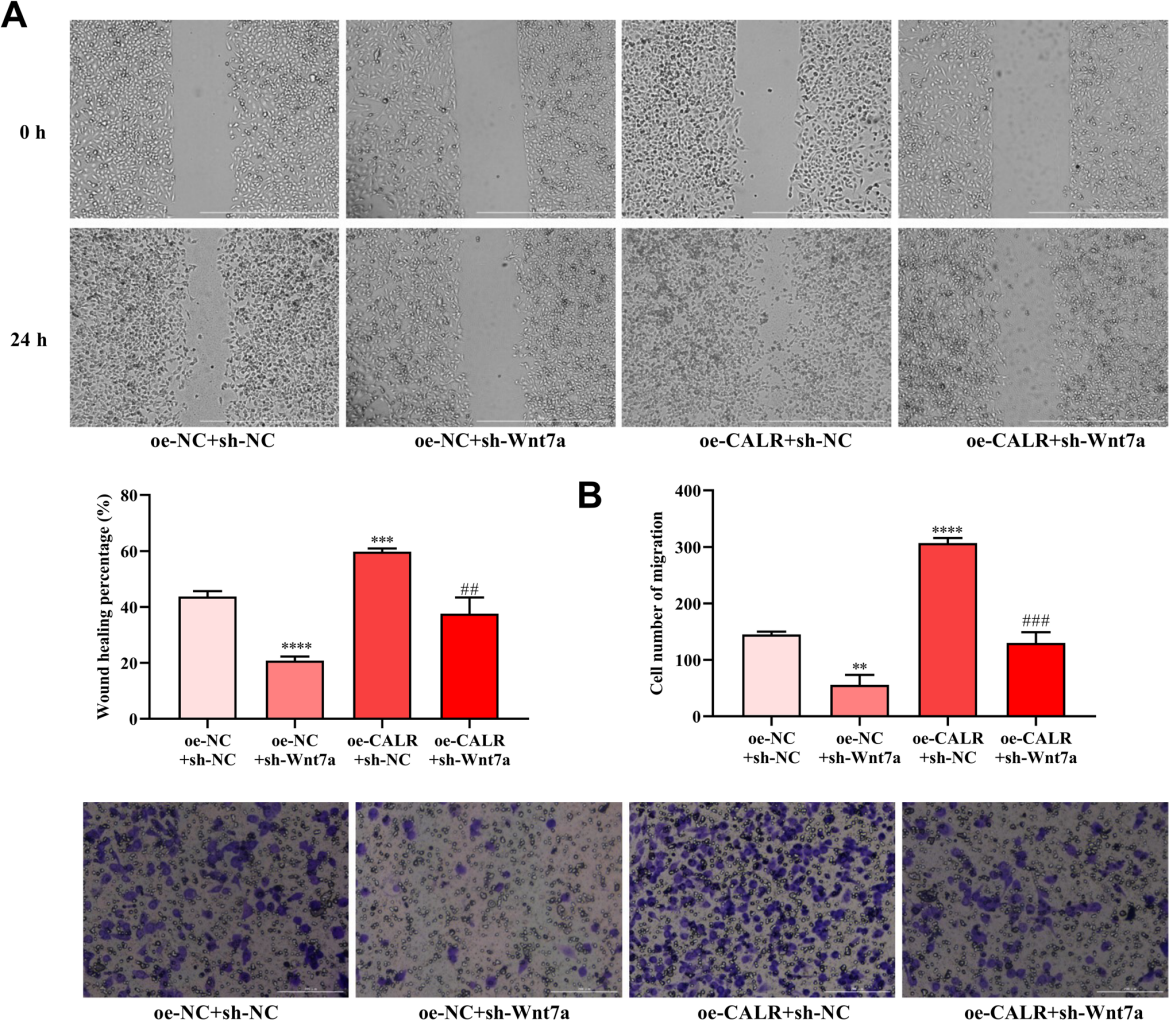
Wnt7a knockdown reversed the effects of CALR overexpression on the migration of HCE-2[50.B1] cells. **A**, **B** The migration of HCE-2[50.B1] cells with oe-CALR and sh-Wnt7a treatment was measured by wound healing and Transwell assays. **P < 0.01, ***P < 0.001, ****P < 0.0001, compared with oe-NC + sh-NC group. ##*P* < 0.01, ###*P* < 0.001, compared with oe-CALR + sh-NC group

Effect of Wnt7a knockdown on the migration ability of HCE-2[50.B1] cells promoted by CALR overexpression was measured by wound healing and Transwell assays. After co-transfection of sh-Wnt7a, the migration ability and transmembrane number of HCE-2[50.B1] cells with CALR overexpression were decreased (Fig. 6). These indicated that CALR regulated migration of HCE-2[50.B1] cells through Wnt7a.

## Discussion

Corneal epithelial injury refers to the destruction of the function and integrity of the corneal epithelial barrier caused by various factors, resulting in the loss of part or all of the corneal epithelial cell layer [2]. Clinically, it can manifest as diffuse punctate loss or erosion of the corneal epithelium, and in severe cases, it can lead to corneal stromal lesions, affecting visual function and even blindness [19]. The repair of corneal epithelial injury is a very complex process, involving many coordinated events including cell proliferation, migration, differentiation and death [20, 21]. The underlying mechanisms also include the signal-mediated interaction of growth factors, cytokines and extracellular matrix at the wound site to rebuild the integrity of epithelial cells and restore corneal homeostasis [20, 21]. However, the molecular regulation mechanism of this process is still unclear. Therefore, a better understanding of molecular mechanisms of corneal injury repair will be beneficial for promoting clinical diagnosis and treatment of corneal epithelial repair-related diseases.

Here, CALR expression was increased in mice corneal epithelial injury repair versus normal corneal epithelial. This indicated that CALR might involve in the process of corneal injury repair. CALR promotes wound healing through various biological effects, including cell proliferation, migration, fibrosis, and extracellular matrix production [11]. CALR can promote skin wound repair by inducing cell proliferation (such as fibroblasts, and microvascular endothelial cells) and migration (such as keratinocytes and fibroblasts) [22]. Mishra et al. found that exogenous CALR-treatment promoted corneal wound healing and reduced fibrosis and angiogenesis in rabbit corneal injury model, and promoted human corneal epithelial cells and keratocytes proliferation and migration [13]. Here, oe-CALR was transfected into HCE-2[50.B1] cells to promote its overexpression. Cell function assays found that CALR overexpression could promote HCE-2[50.B1] cells proliferation and growth. Flow cytometry further showed that CALR overexpression caused an increase in the number of G0/G1 and S phase cells, thereby promoting cell proliferation. In addition, the migration ability and cell senescence of HCE-2[50.B1] cells were promoted and inhibited by CALR overexpression, respectively.

According to HitPredict database prediction, this study found that Wnt7a was one of potential interacting proteins of CALR. CALR can process Wnt7a by folding and asparagine glycosylation modification on the endoplasmic reticulum, which may affect the retention/departure of Wnt7a from the endoplasmic reticulum, thus affecting the Wnt pathway and its downstream [23]. Co-immunoprecipitation confirmed the interaction between CALR and Wnt7a. The interaction between CALR and Wnt7a could activate the downstream β-catenin signaling pathway. In order to confirm whether the regulation of CALR on HCE-2[50.B1] cells proliferation and migration through Wnt7a, oe-CALR and sh-Wnt7a plasmid vectors were co-transfected into HCE-2[50.B1] cells. Wnt7a has been confirmed to be upregulated and promote corneal epithelial cell proliferation in injured corneal tissue [17, 18, 24]. Here, Wnt7a knockdown reversed the effect of CALR overexpression on the proliferation, senescence and migration of HCE-2[50.B1] cells. These results indicated that CALR promoted corneal epithelial wound healing through Wnt7a. This might be that CALR ensures the correct folding and transport assembly of the Wnt7 precursor, thereby increasing the secretion of Wnt7a and leading to an increase in the expression of β-catenin downstream of Wnt7a [23, 25]. Under normal circumstances, β-catenin is degraded in the absence of a Wnt stimulus by the destruction complex composed of AXIN, APC, GSK-3, CK1 and β-TrCP [26]. The increase of wnt7a binds to the receptor Frizzled on the cell membrane and the co-receptor low-density lipoprotein reception-related protein to activate the dishevelled protein and binds to AXIN and GSK-3β proteins, resulting in the decomposition of the degradation complex [27]. After the degradation complex is destroyed, the stably accumulated β-catenin in the cytoplasm enters the nucleus and binds to the LEF/TCF transcription factor family, initiating the transcription of downstream target genes, such as c-myc and Cyclin D1 [28]. Ultimately, it participates in physiological activities such as cell proliferation and differentiation, promoting the repair and healing of the cornea. However, whether CALR and Wnt7a proteins are tightly binding or transient interaction, and how the complex regulates the expression of other genes or signaling pathway in corneal epithelial cells and its specific mechanism need to be improved in future experiments.

## Conclusion

In conclusion, CALR promotes proliferation and migration, inhibited senescence of HCE-2[50.B1] cells through Wnt7a, thus promoting corneal epithelial wound healing. This study deepens understanding of corneal injury repair mechanism, and provides new ideas and therapeutic targets for clinical diagnosis and treatment of corneal epithelial repair-related diseases in humans.

## Supplementary Information

Below is the link to the electronic supplementary material.

Supplementary file1 (TIF 372 KB)

Supplementary file2 (TIF 215 KB)

Supplementary file3 (TIF 1222 KB)

Supplementary file4 (TIF 402 KB)

Supplementary file5 (TIF 225 KB)

Supplementary file6 (TIF 1511 KB)

Supplementary file7 (JPG 168 KB)

Supplementary file8 (JPG 197 KB)

Supplementary file9 (PNG 2618 KB)

Supplementary file10 (PNG 2618 KB)

Supplementary file11 (PNG 4548 KB)

Supplementary file12 (PNG 4646 KB)

Supplementary file13 (PNG 1425 KB)

Supplementary file14 (PNG 1724 KB)

Supplementary file15 (PNG 1420 KB)

Supplementary file16 (PNG 1487 KB)

Supplementary file17 (PNG 3152 KB)

Supplementary file18 (PNG 3003 KB)

Supplementary file19 (TIF 393 KB)

Supplementary file20 (TIF 452 KB)

Supplementary file21 (TIF 782 KB)

Supplementary file22 (TIF 381 KB)

Supplementary file23 (TIF 289 KB)

Supplementary file24 (TIF 3123 KB)

Supplementary file25 (TIF 120 KB)

Supplementary file26 (TIF 425 KB)

Supplementary file27 (TIF 285 KB)

Supplementary file28 (TIF 272 KB)

Supplementary file29 (TIF 347 KB)

Supplementary file30 (TIF 3790 KB)

Supplementary file31 (TIF 2077 KB)

Supplementary file32 (TIF 87 KB)

Supplementary file33 (TIF 114 KB)

Supplementary file34 (TIF 362 KB)

Supplementary file35 (TIF 136 KB)

Supplementary file36 (TIF 126 KB)

Supplementary file37 (TIF 315 KB)

Supplementary file38 (JPG 178 KB)

Supplementary file39 (JPG 194 KB)

Supplementary file40 (JPG 200 KB)

Supplementary file41 (JPG 158 KB)

Supplementary file42 (PNG 2618 KB)

Supplementary file43 (PNG 2618 KB)

Supplementary file44 (PNG 2618 KB)

Supplementary file45 (PNG 2618 KB)

Supplementary file46 (PNG 4514 KB)

Supplementary file47 (PNG 4526 KB)

Supplementary file48 (PNG 4393 KB)

Supplementary file49 (PNG 4643 KB)

Supplementary file50 (PNG 1452 KB)

Supplementary file51 (PNG 1618 KB)

Supplementary file52 (PNG 1377 KB)

Supplementary file53 (PNG 1519 KB)

Supplementary file54 (PNG 1410 KB)

Supplementary file55 (PNG 1527 KB)

Supplementary file56 (PNG 1396 KB)

Supplementary file57 (PNG 1476 KB)

Supplementary file58 (PNG 3163 KB)

Supplementary file59 (PNG 2888 KB)

Supplementary file60 (PNG 2924 KB)

Supplementary file61 (PNG 2882 KB)

## Data Availability

The datasets used and/or analysed during the current study available from the corresponding author on reasonable request.

## References

[1] Xu W et al (2022) PCL scaffold combined with rat tail collagen type I to reduce keratocyte differentiation and prevent corneal stroma fibrosis after injury. Exp Eye Res 217:108936 35093391 10.1016/j.exer.2022.108936

[2] Wilson SE (2020) Corneal wound healing. Exp Eye Res 197:108089 32553485 10.1016/j.exer.2020.108089 PMC7483425

[3] Sugioka K et al (2021) The fibrinolytic system in the cornea: A key regulator of corneal wound healing and biological defense. Exp Eye Res 204:108459 33493476 10.1016/j.exer.2021.108459

[4] Tarvestad-Laise KE, Ceresa BP (2023) Modulating growth factor receptor signaling to promote corneal epithelial homeostasis. Cells 12(23):2730 38067157 10.3390/cells12232730 PMC10706396

[5] Akowuah PK et al (2021) An Epithelial Abrasion Model for Studying Corneal Wound Healing. J Vis Exp. 10.3791/63112 35037655 10.3791/63112 PMC9102860

[6] Wagoner MD (1997) Chemical injuries of the eye: current concepts in pathophysiology and therapy. Surv Ophthalmol 41(4):275–313 9104767 10.1016/s0039-6257(96)00007-0

[7] Agellon LB, Michalak M (2021) A view of the endoplasmic reticulum through the calreticulin lens. Prog Mol Subcell Biol 59:1–11 34050859 10.1007/978-3-030-67696-4_1

[8] Sawaya AP et al (2023) Calreticulin: a multifunctional protein with potential therapeutic applications for chronic wounds. Front Med (Lausanne) 10:1207538 37692787 10.3389/fmed.2023.1207538 PMC10484228

[9] Palacon MP et al (2023) Calreticulin expression in human carcinomas: a systematic review and meta-analysis. Asian Pac J Cancer Prev 24(9):2929 37774043 10.31557/APJCP.2023.24.9.2929 PMC10762747

[10] Zhang M et al (2023) Calreticulin as a marker and therapeutic target for cancer. Clin Exp Med 23(5):1393–1404 36335525 10.1007/s10238-022-00937-7

[11] Gold LI et al (2006) Overview of the role for calreticulin in the enhancement of wound healing through multiple biological effects. J Investig Dermatol Symp Proc 11(1):57–65 17069011 10.1038/sj.jidsymp.5650011

[12] Greives MR et al (2012) Exogenous calreticulin improves diabetic wound healing. Wound Repair Regen 20(5):715–730 22985041 10.1111/j.1524-475X.2012.00822.x

[13] Mishra S et al (2023) Calreticulin accelerates corneal wound closure and mitigates fibrosis: Potential therapeutic applications. J Cell Mol Med 28(5):e18027 37985392 10.1111/jcmm.18027 PMC10902309

[14] Hayat R, Manzoor M, Hussain A (2022) Wnt signaling pathway: A comprehensive review. Cell Biol Int 46(6):863–877 35297539 10.1002/cbin.11797

[15] Ng LF et al (2019) WNT signaling in disease. Cells 8(8):826 31382613 10.3390/cells8080826 PMC6721652

[16] Weizhuo Q et al (2023) Research progress of Wnt/β-catenin signaling pathway in diseases. Modern Med J 51(09):1337–1345

[17] Lan L et al (2019) Roles of Wnt7a in embryo development, tissue homeostasis, and human diseases. J Cell Biochem 120(11):18588–18598 31271226 10.1002/jcb.29217

[18] Lyu J, Joo C-K (2005) Wnt-7a up-regulates matrix metalloproteinase-12 expression and promotes cell proliferation in corneal epithelial cells during wound healing. J Biol Chem 280(22):21653–21660 15802269 10.1074/jbc.M500374200

[19] Sun CC et al (2021) Cisd2 plays an essential role in corneal epithelial regeneration. EBioMedicine 73:103654 34740104 10.1016/j.ebiom.2021.103654 PMC8577409

[20] Sun CC et al (2023) Targeting Ca(2+)-dependent pathways to promote corneal epithelial wound healing induced by CISD2 deficiency. Cell Signal 109:110755 37315750 10.1016/j.cellsig.2023.110755

[21] Wang M et al (2022) The therapeutic roles of recombinant Hsp90α on cornea epithelial injury. Invest Ophthalmol Vis Sci 63(2):30 35201262 10.1167/iovs.63.2.30 PMC8883155

[22] Nanney LB et al (2008) Calreticulin enhances porcine wound repair by diverse biological effects. Am J Pathol 173(3):610–630 18753412 10.2353/ajpath.2008.071027 PMC2527067

[23] Qi X et al (2023) Molecular basis of Wnt biogenesis, secretion, and Wnt7-specific signaling. Cell 186(23):5028–5040 37852257 10.1016/j.cell.2023.09.021 PMC10841698

[24] Lyu J, Joo CK (2006) Expression of Wnt and MMP in epithelial cells during corneal wound healing. Cornea 25(10 Suppl 1):S24–S28 17001188 10.1097/01.ico.0000247209.01262.4e

[25] Manukjan N et al (2024) Wnt7a decreases brain endothelial barrier function via β-catenin activation. Mol Neurobiol 61(7):4854–4867 38147228 10.1007/s12035-023-03872-0 PMC11236883

[26] Stamos JL, Weis WI (2013) The β-catenin destruction complex. Cold Spring Harb Perspect Biol 5(1):a007898 23169527 10.1101/cshperspect.a007898 PMC3579403

[27] Yu M et al (2020) The research progress of Wnt signaling pathway in self-renewal of hematopoietic stem cells. Chin Bull Life Sci 32(05):413–423

[28] Reyes M et al (2020) Wnt/β-catenin signaling in oral carcinogenesis. Int J Mol Sci 21(13):4682 32630122 10.3390/ijms21134682 PMC7369957

