# Supplementary figures and images for: CALR promotes corneal epithelial cell proliferation and migration through Wnt7a
Source: Mol Biol Rep. 2025 Jul 16;52(1):714. doi: 10.1007/s11033-025-10810-x (PMC12267377; doi:10.1007/s11033-025-10810-x)

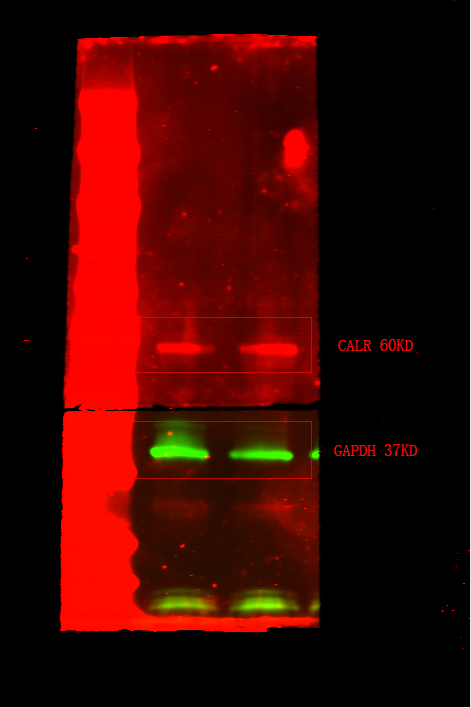

Supplement: Supplementary file 3 — Supplementary file3 (TIF 1222 KB) [file 11033_2025_10810_MOESM3_ESM.tif]

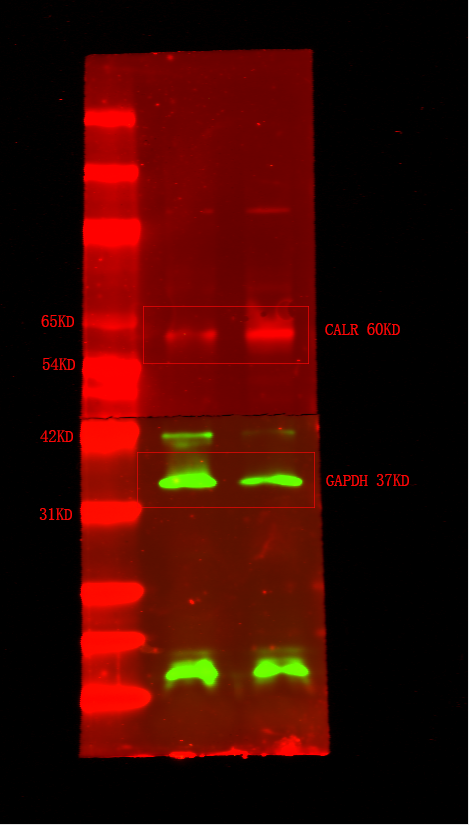

Supplement: Supplementary file 6 — Supplementary file6 (TIF 1511 KB) [file 11033_2025_10810_MOESM6_ESM.tif]

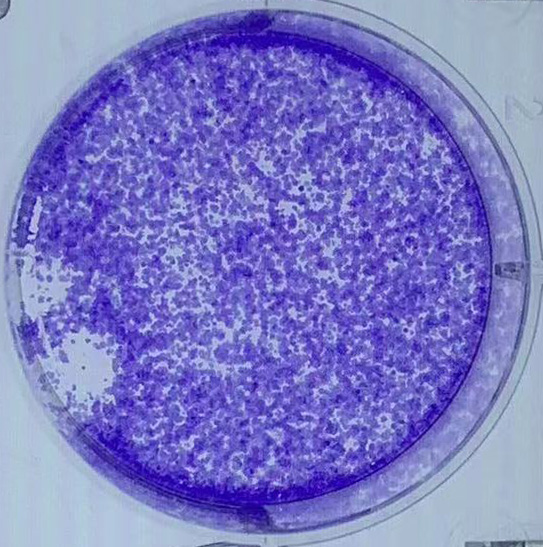

Supplement: Supplementary file 7 — Supplementary file7 (JPG 168 KB) [file 11033_2025_10810_MOESM7_ESM.jpg]

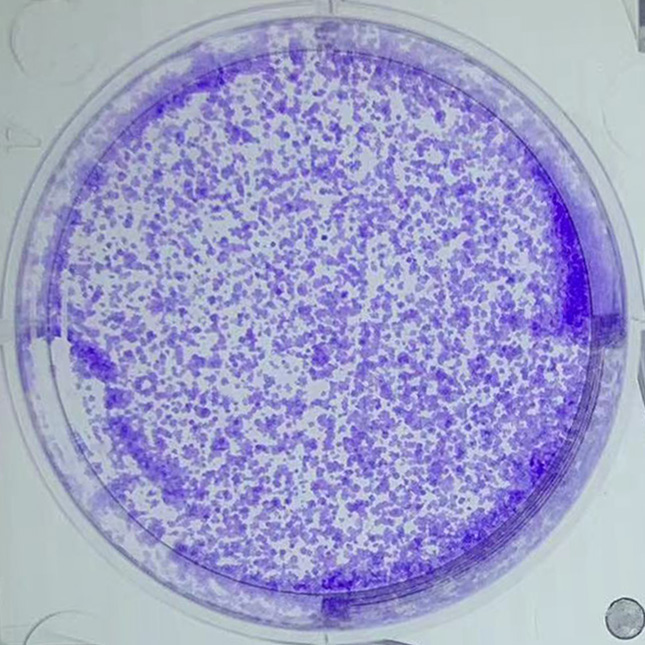

Supplement: Supplementary file 8 — Supplementary file8 (JPG 197 KB) [file 11033_2025_10810_MOESM8_ESM.jpg]

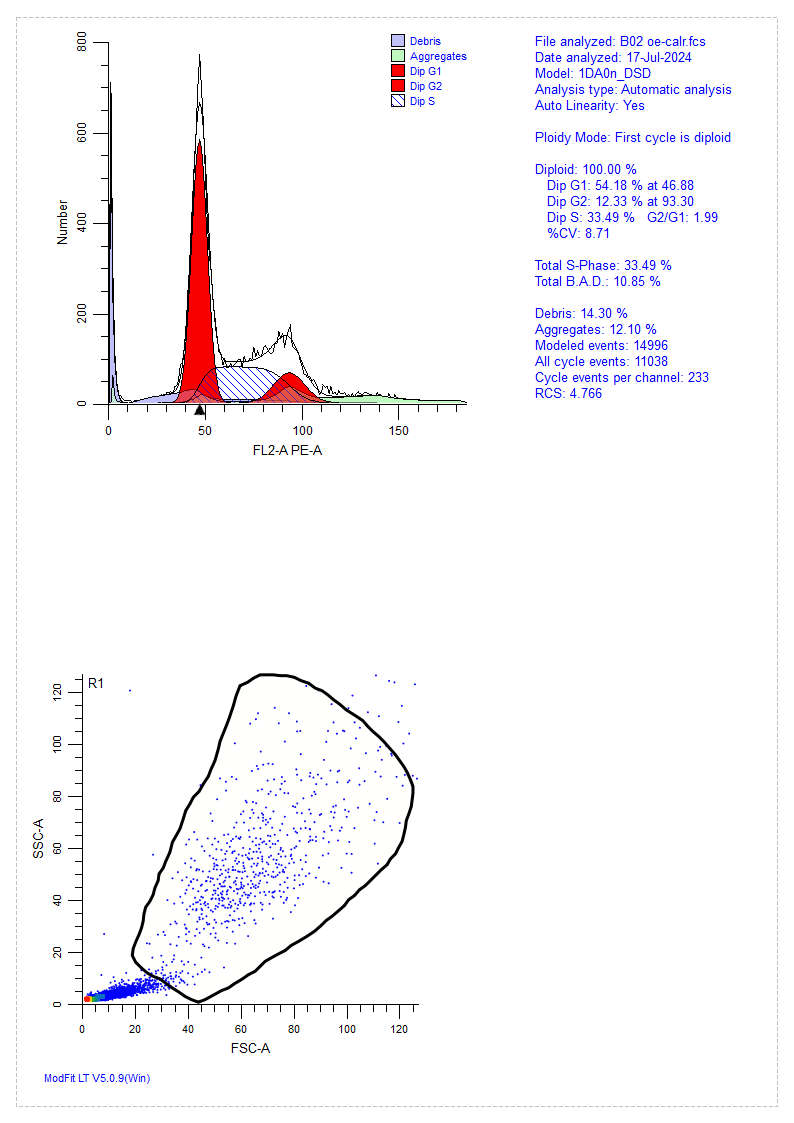

Supplement: Supplementary file 9 — Supplementary file9 (PNG 2618 KB) [file 11033_2025_10810_MOESM9_ESM.png]

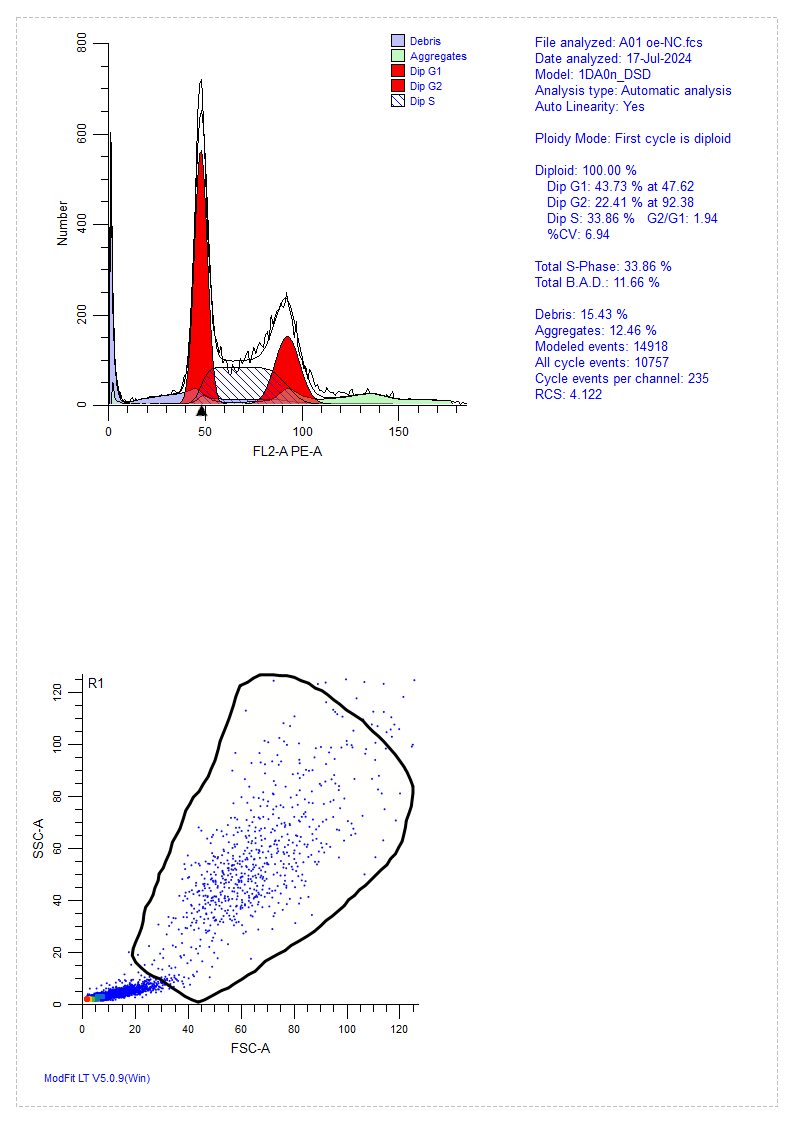

Supplement: Supplementary file 10 — Supplementary file10 (PNG 2618 KB) [file 11033_2025_10810_MOESM10_ESM.png]

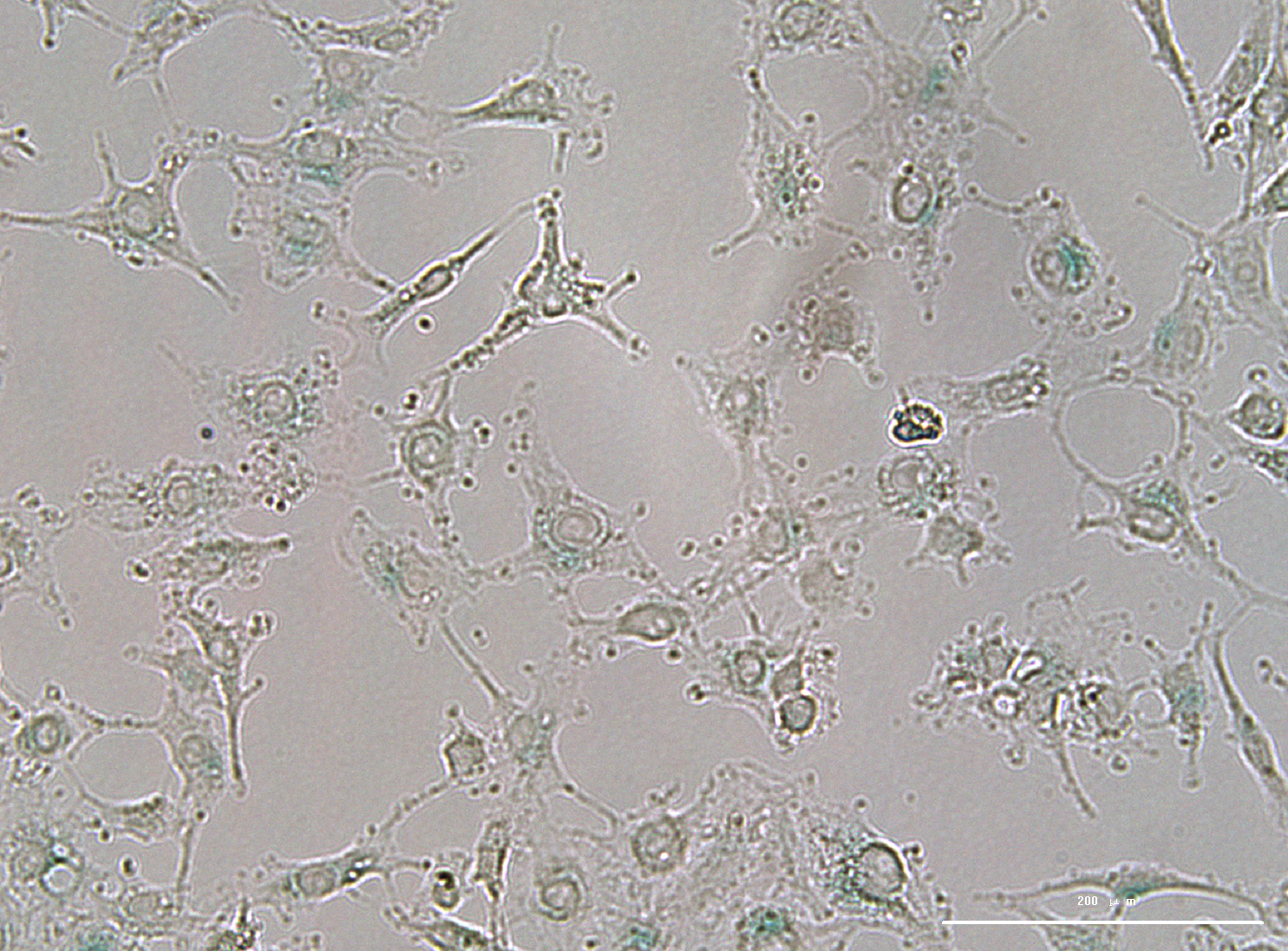

Supplement: Supplementary file 11 — Supplementary file11 (PNG 4548 KB) [file 11033_2025_10810_MOESM11_ESM.png]

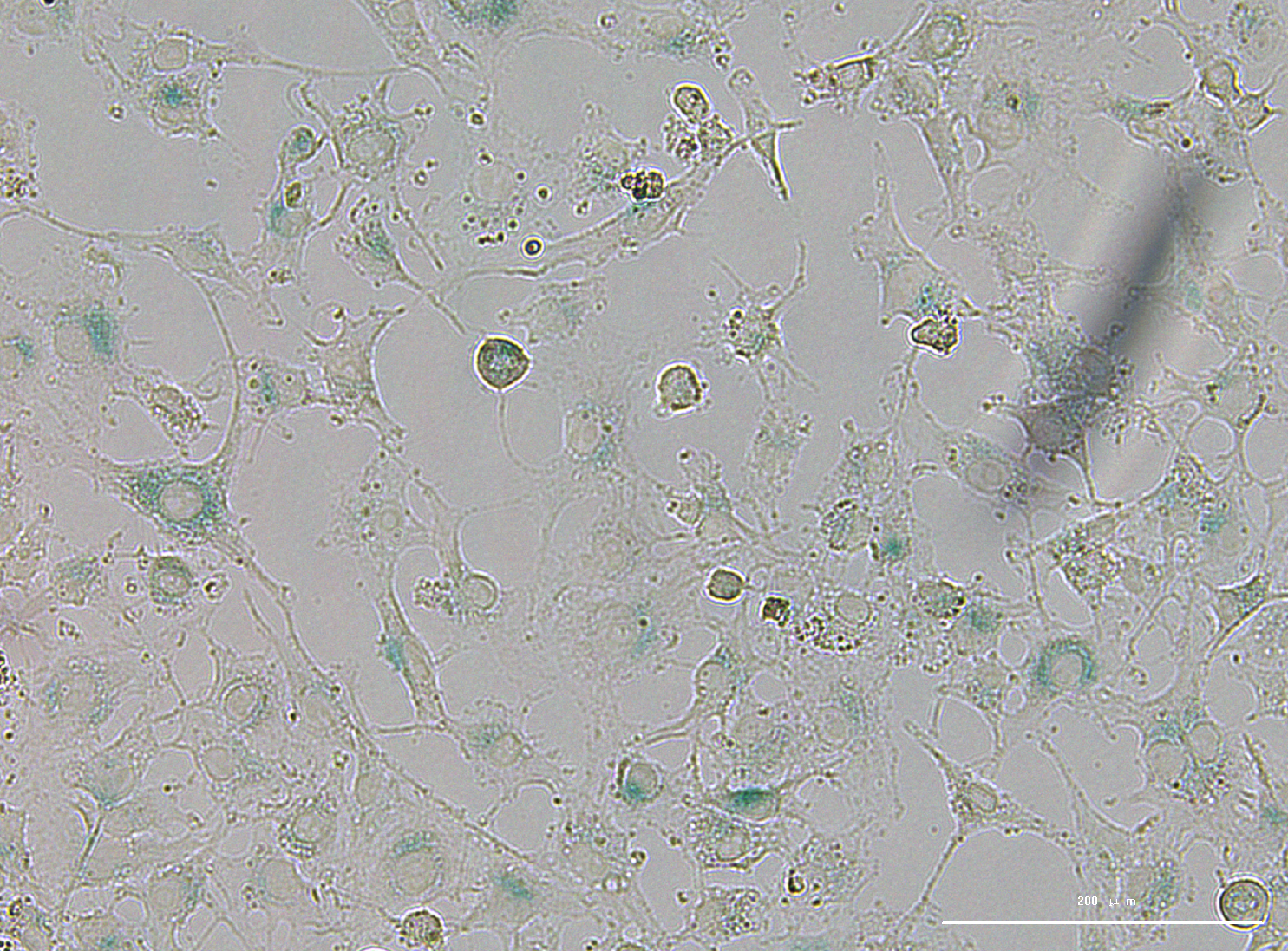

Supplement: Supplementary file 12 — Supplementary file12 (PNG 4646 KB) [file 11033_2025_10810_MOESM12_ESM.png]

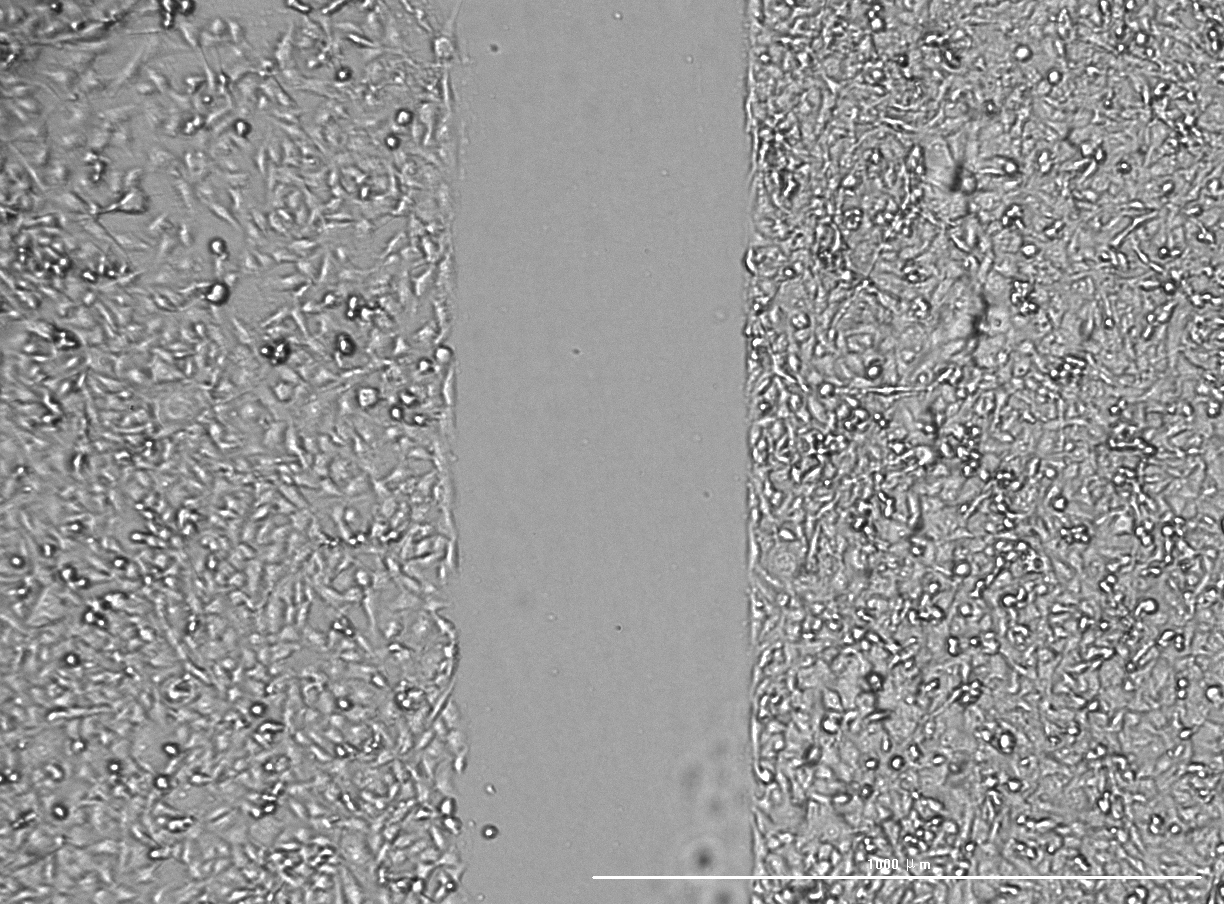

Supplement: Supplementary file 13 — Supplementary file13 (PNG 1425 KB) [file 11033_2025_10810_MOESM13_ESM.png]

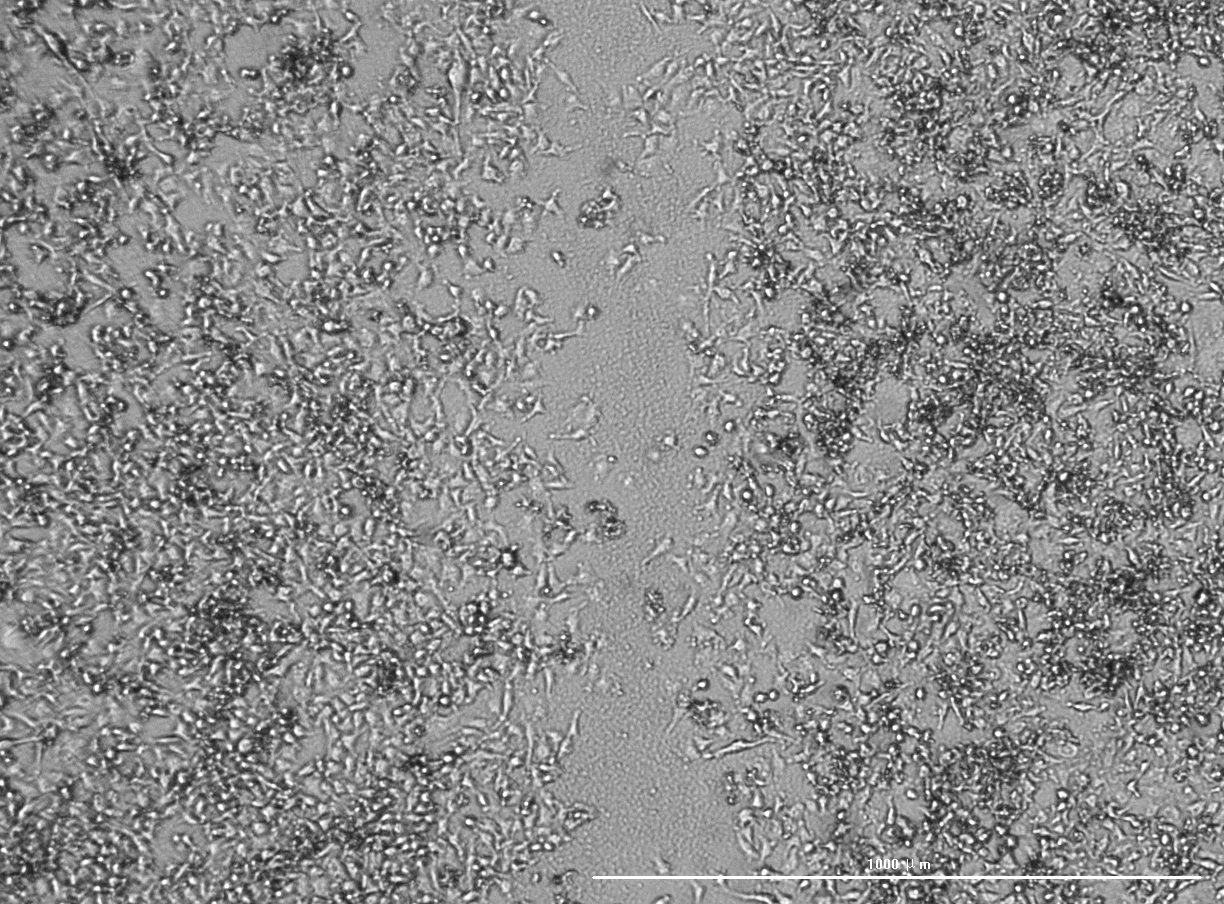

Supplement: Supplementary file 14 — Supplementary file14 (PNG 1724 KB) [file 11033_2025_10810_MOESM14_ESM.png]

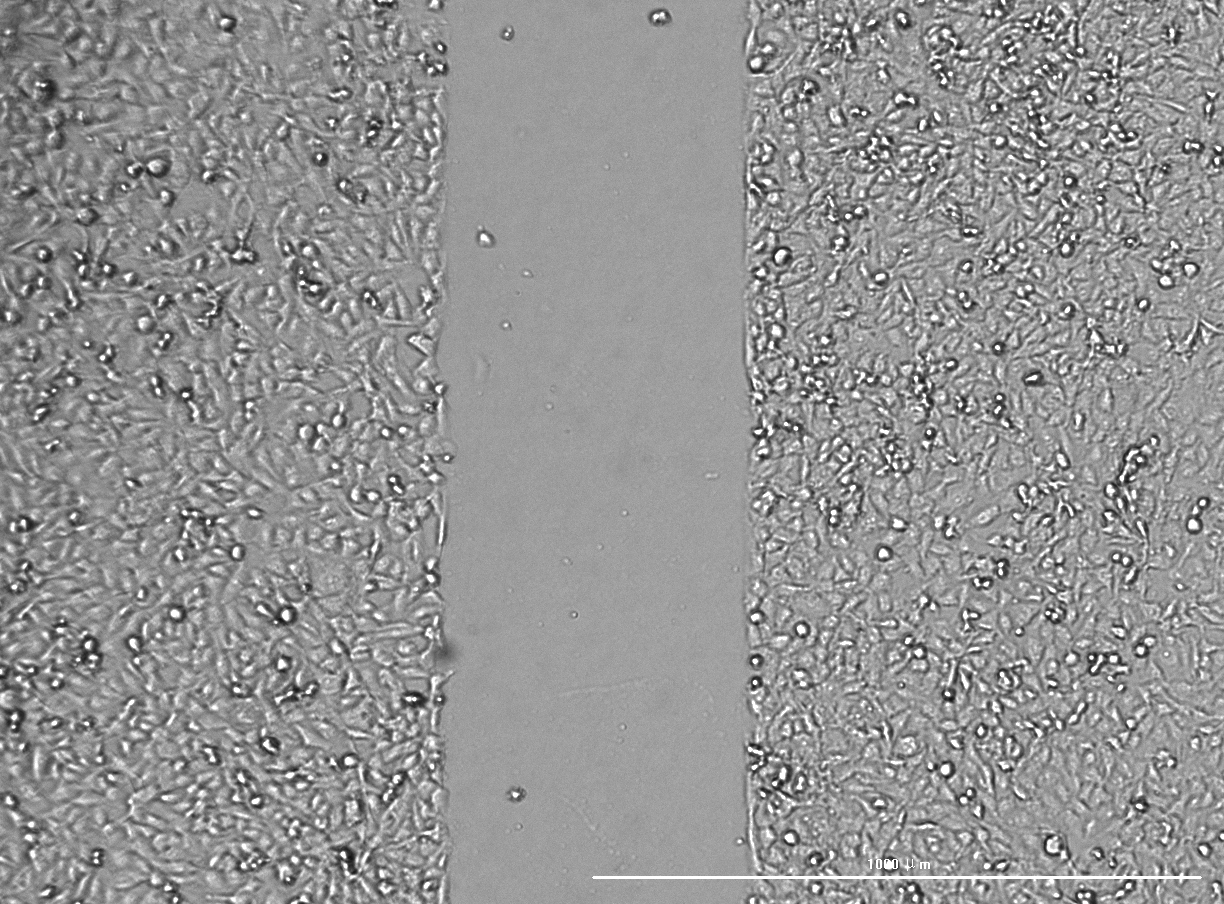

Supplement: Supplementary file 15 — Supplementary file15 (PNG 1420 KB) [file 11033_2025_10810_MOESM15_ESM.png]

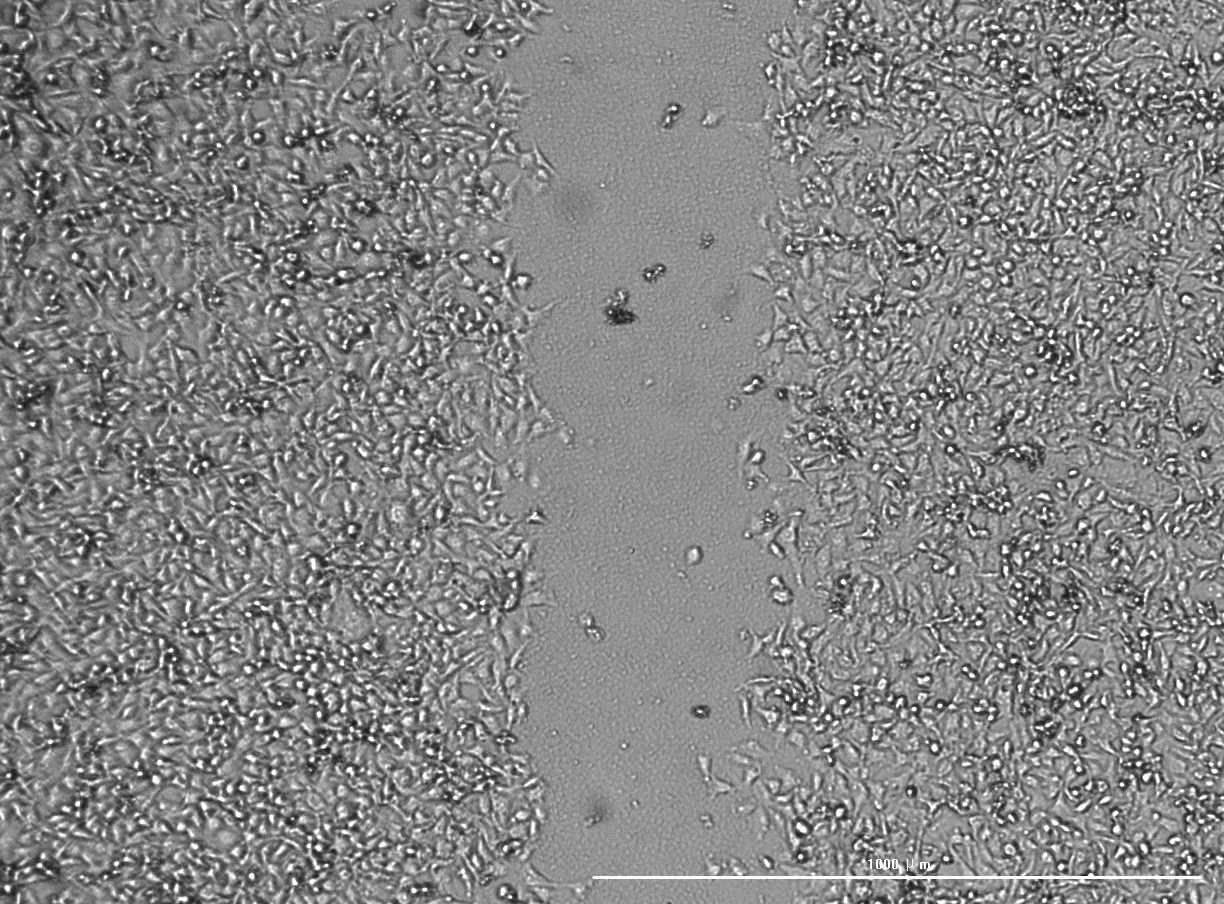

Supplement: Supplementary file 16 — Supplementary file16 (PNG 1487 KB) [file 11033_2025_10810_MOESM16_ESM.png]

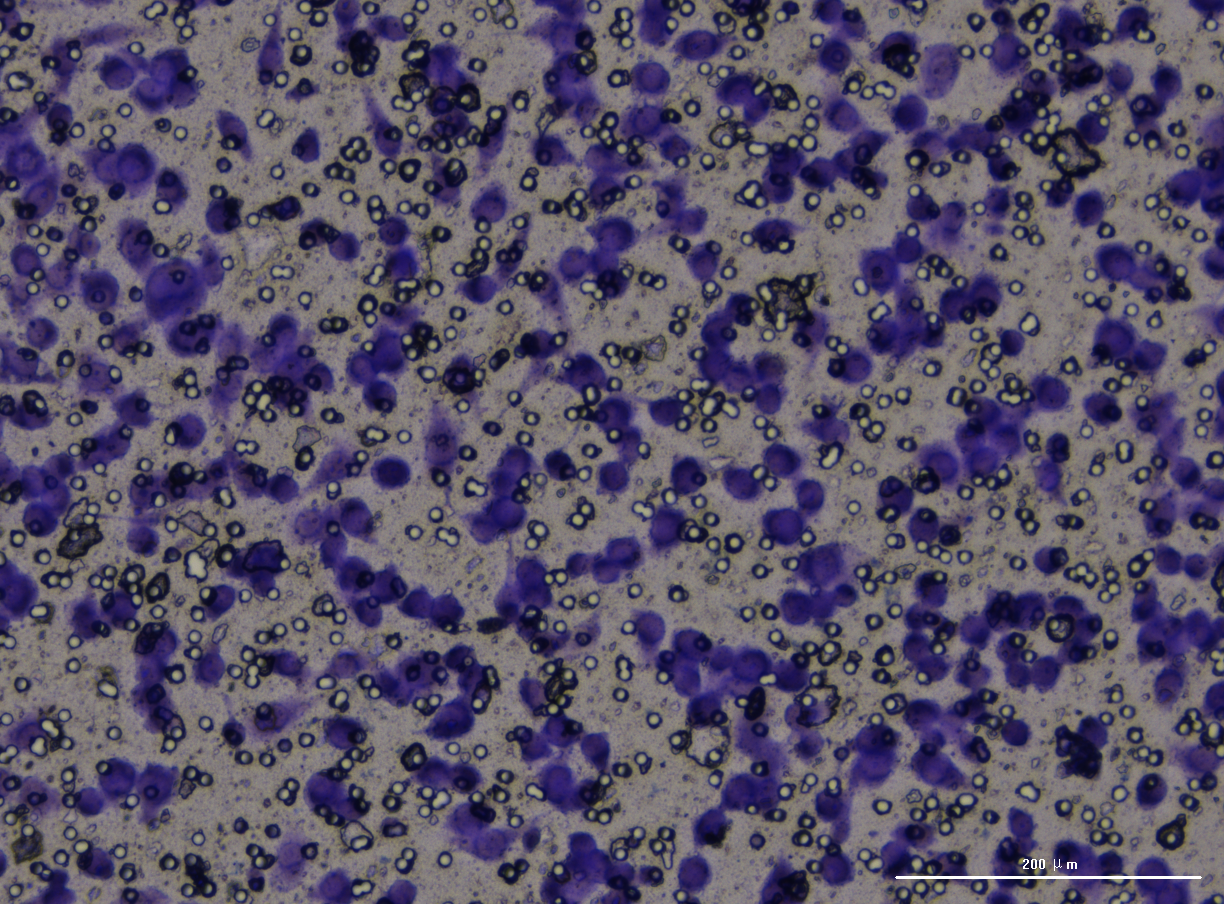

Supplement: Supplementary file 17 — Supplementary file17 (PNG 3152 KB) [file 11033_2025_10810_MOESM17_ESM.png]

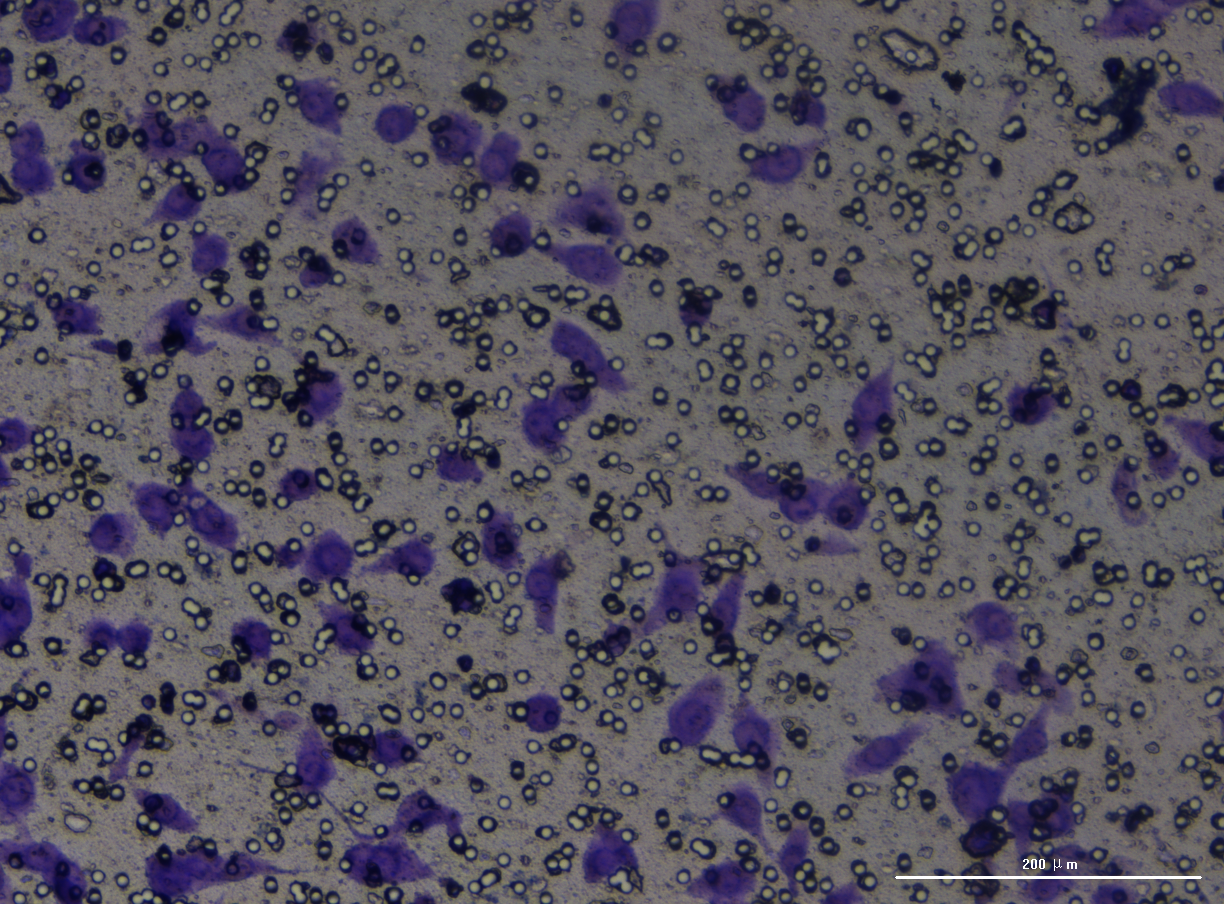

Supplement: Supplementary file 18 — Supplementary file18 (PNG 3003 KB) [file 11033_2025_10810_MOESM18_ESM.png]

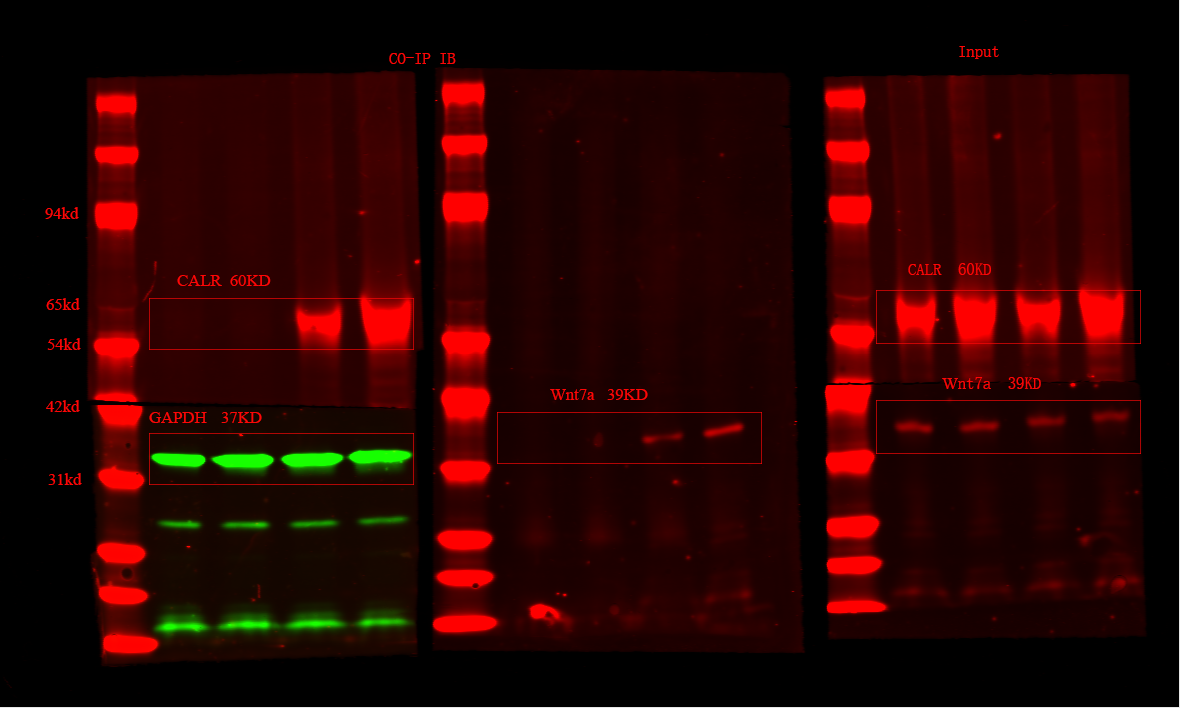

Supplement: Supplementary file 24 — Supplementary file24 (TIF 3123 KB) [file 11033_2025_10810_MOESM24_ESM.tif]

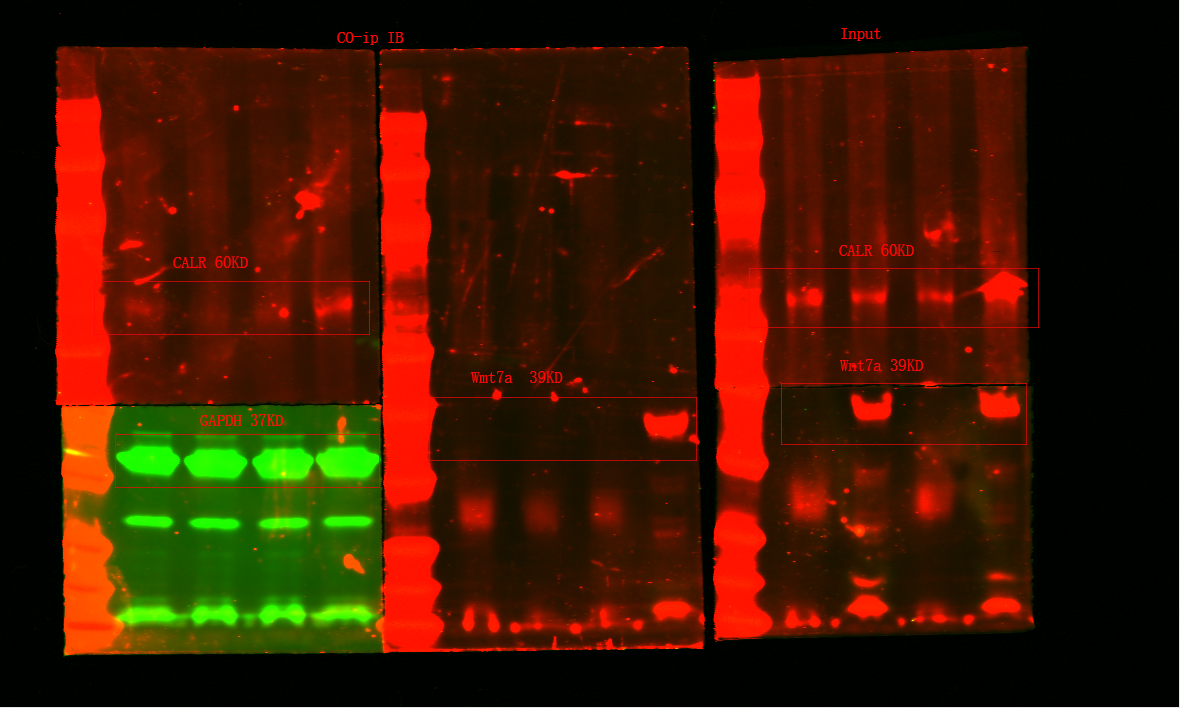

Supplement: Supplementary file 30 — Supplementary file30 (TIF 3790 KB) [file 11033_2025_10810_MOESM30_ESM.tif]

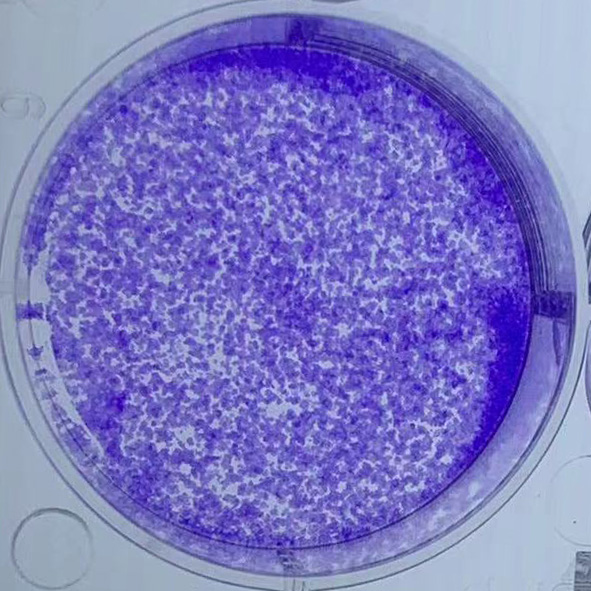

Supplement: Supplementary file 38 — Supplementary file38 (JPG 178 KB) [file 11033_2025_10810_MOESM38_ESM.jpg]

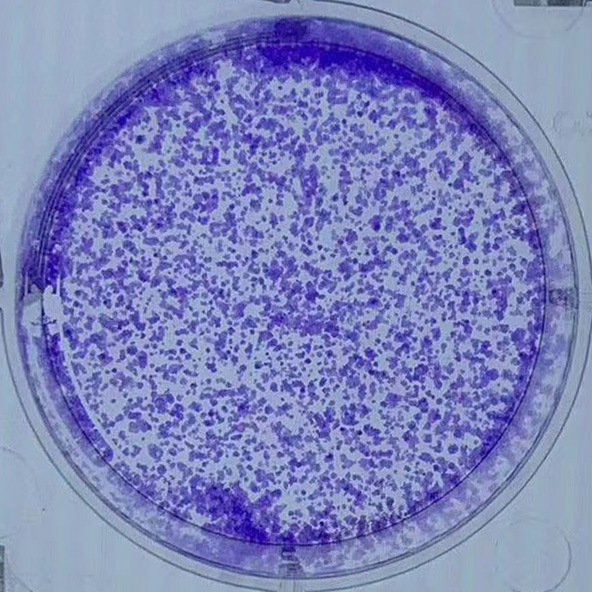

Supplement: Supplementary file 39 — Supplementary file39 (JPG 194 KB) [file 11033_2025_10810_MOESM39_ESM.jpg]

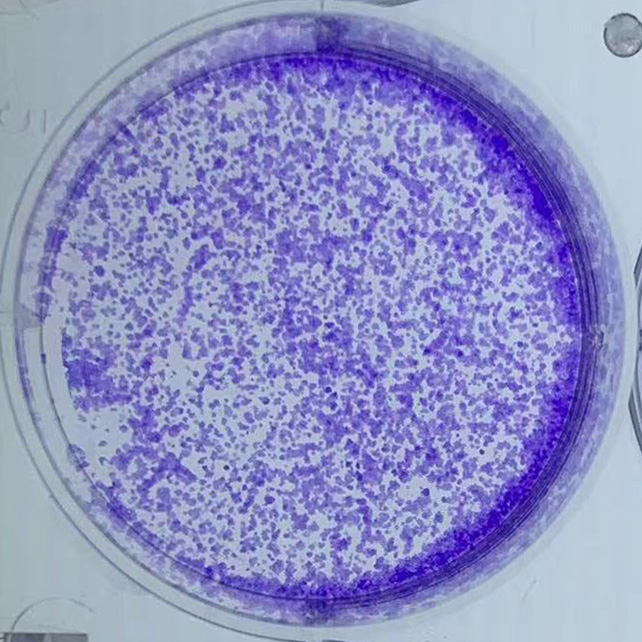

Supplement: Supplementary file 40 — Supplementary file40 (JPG 200 KB) [file 11033_2025_10810_MOESM40_ESM.jpg]

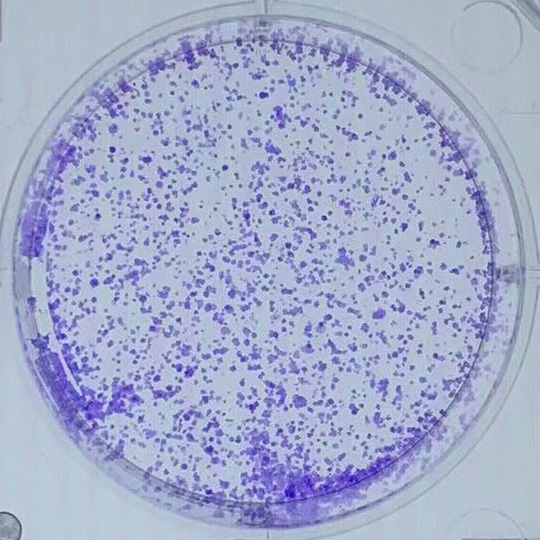

Supplement: Supplementary file 41 — Supplementary file41 (JPG 158 KB) [file 11033_2025_10810_MOESM41_ESM.jpg]

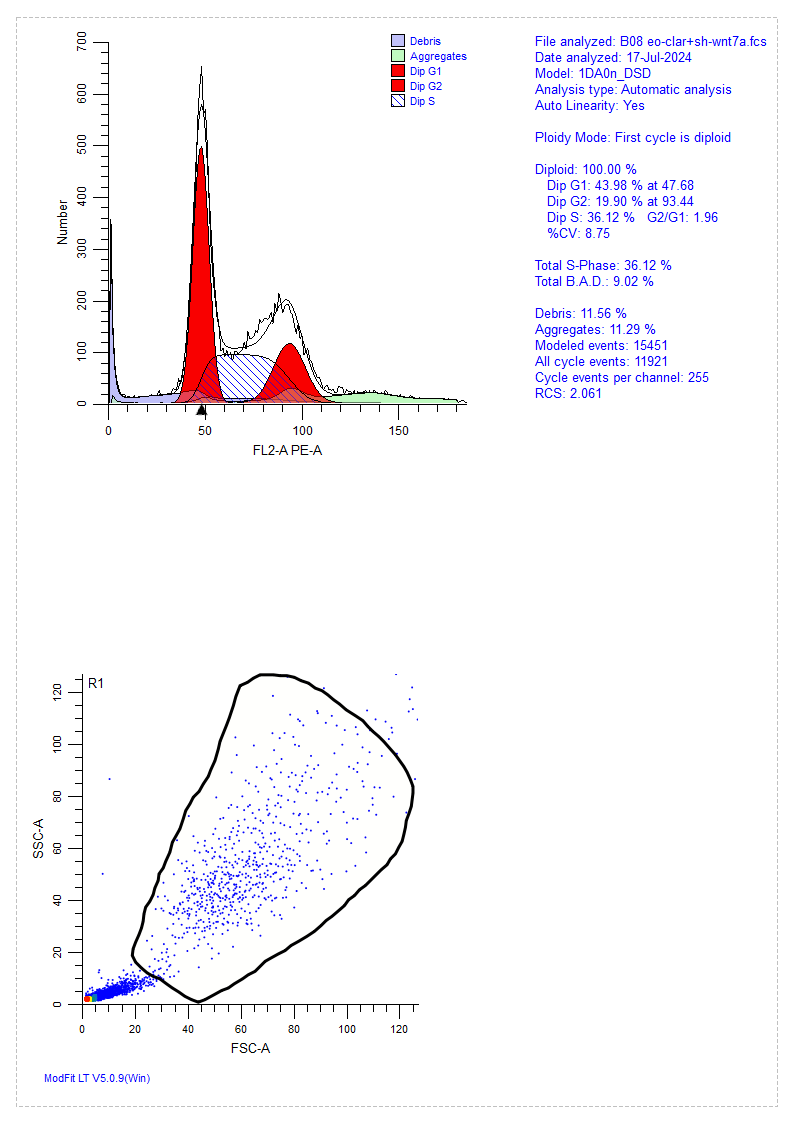

Supplement: Supplementary file 42 — Supplementary file42 (PNG 2618 KB) [file 11033_2025_10810_MOESM42_ESM.png]

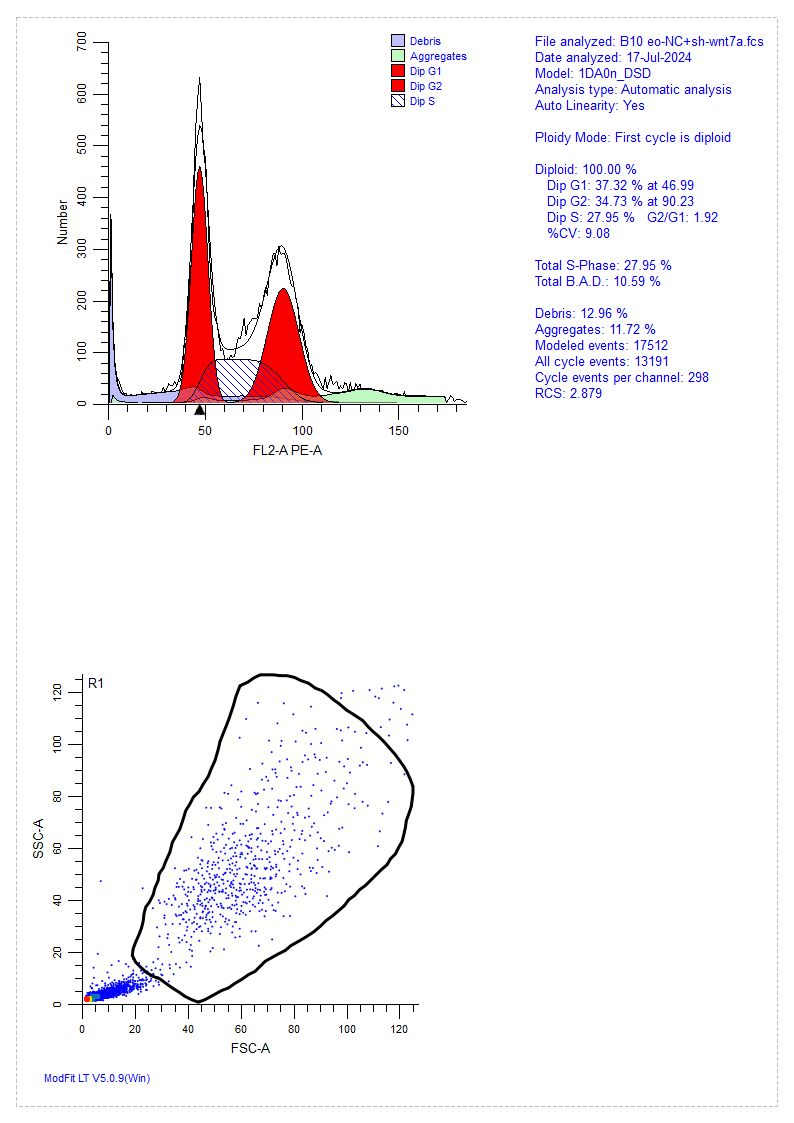

Supplement: Supplementary file 43 — Supplementary file43 (PNG 2618 KB) [file 11033_2025_10810_MOESM43_ESM.png]

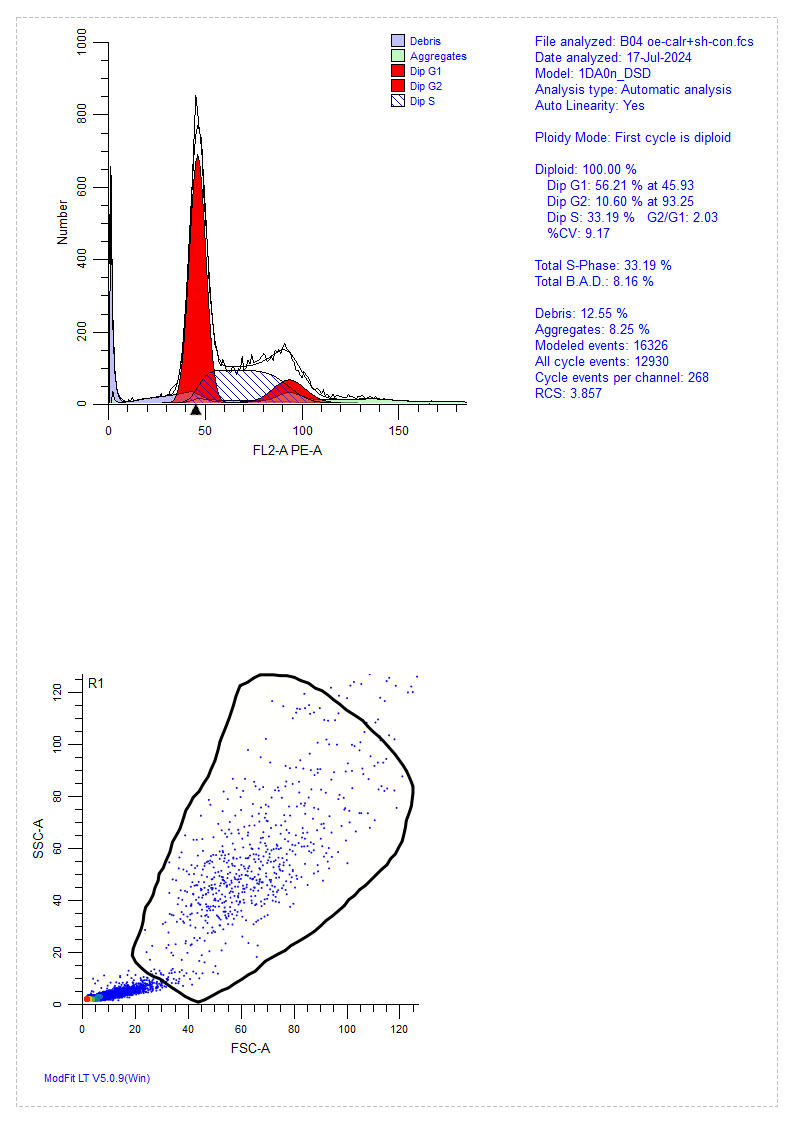

Supplement: Supplementary file 44 — Supplementary file44 (PNG 2618 KB) [file 11033_2025_10810_MOESM44_ESM.png]

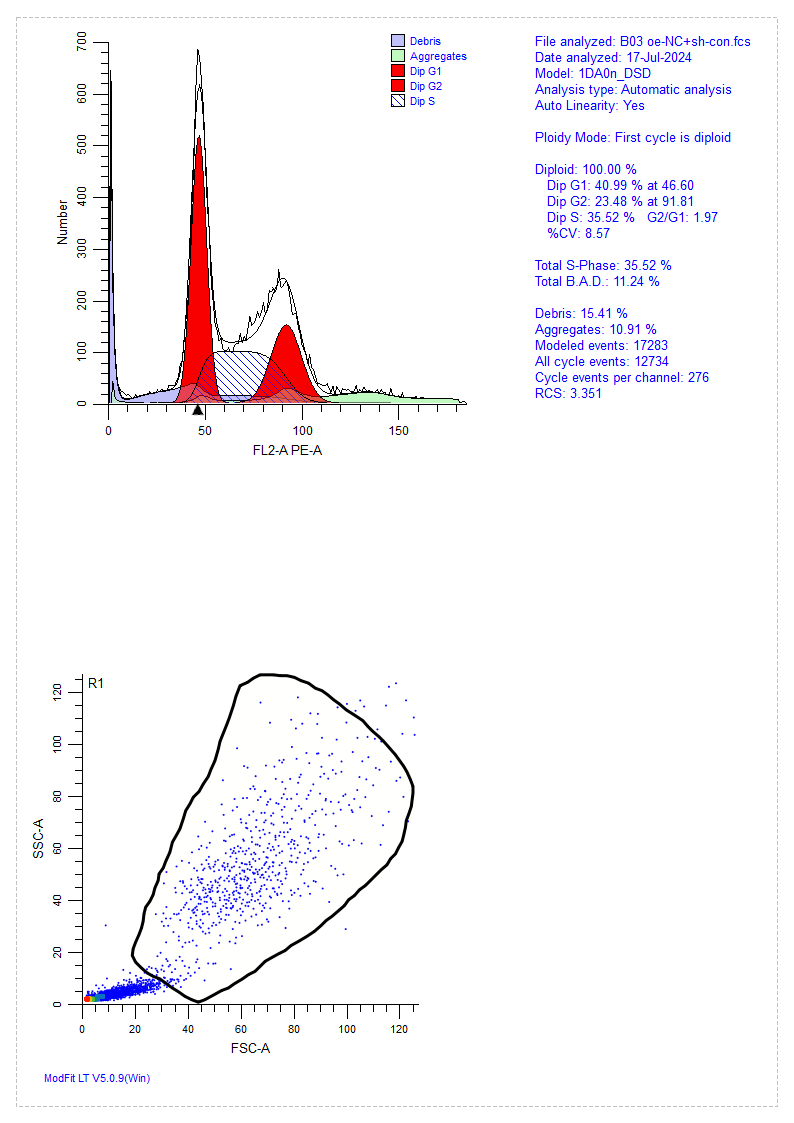

Supplement: Supplementary file 45 — Supplementary file45 (PNG 2618 KB) [file 11033_2025_10810_MOESM45_ESM.png]

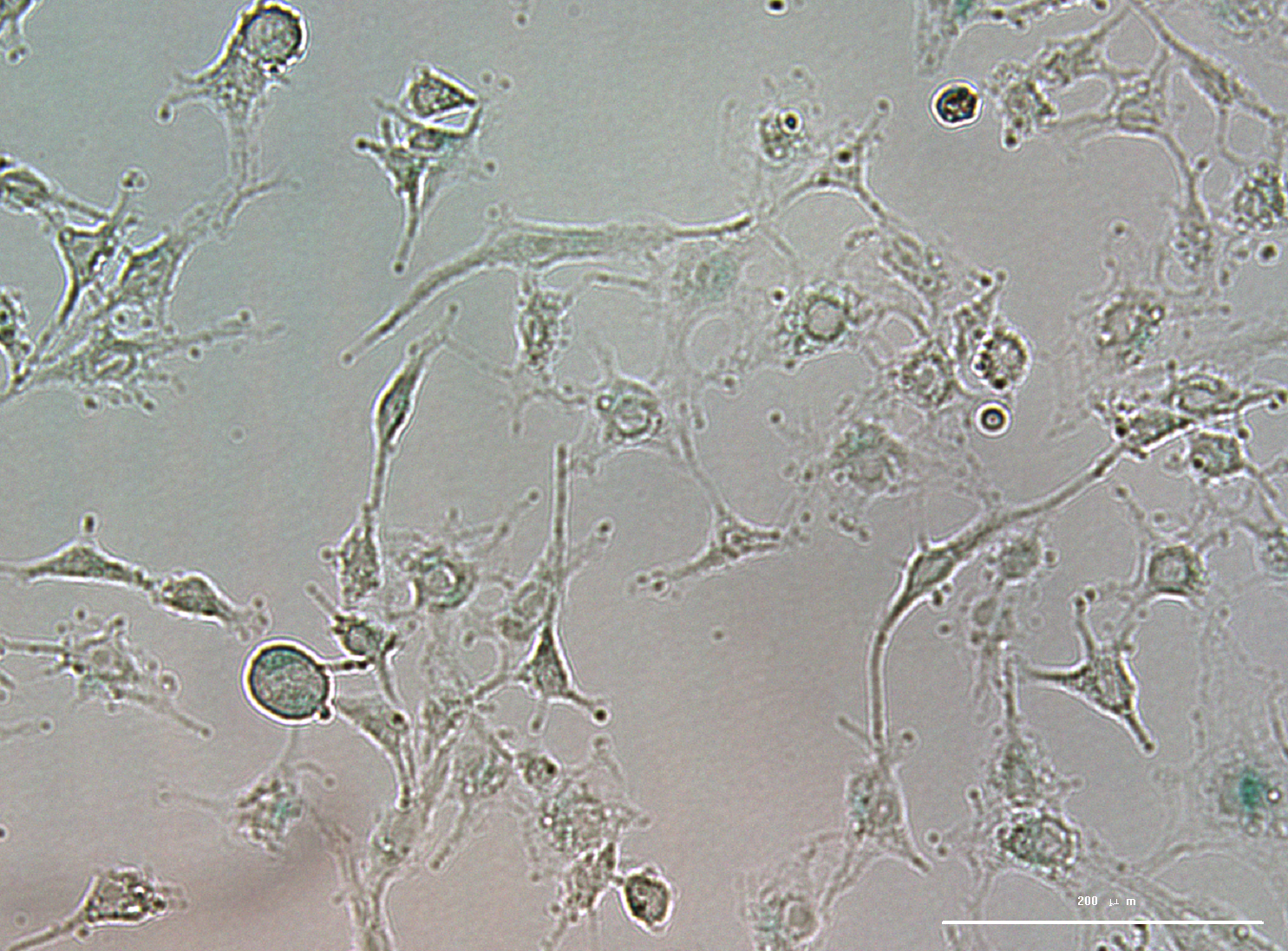

Supplement: Supplementary file 46 — Supplementary file46 (PNG 4514 KB) [file 11033_2025_10810_MOESM46_ESM.png]

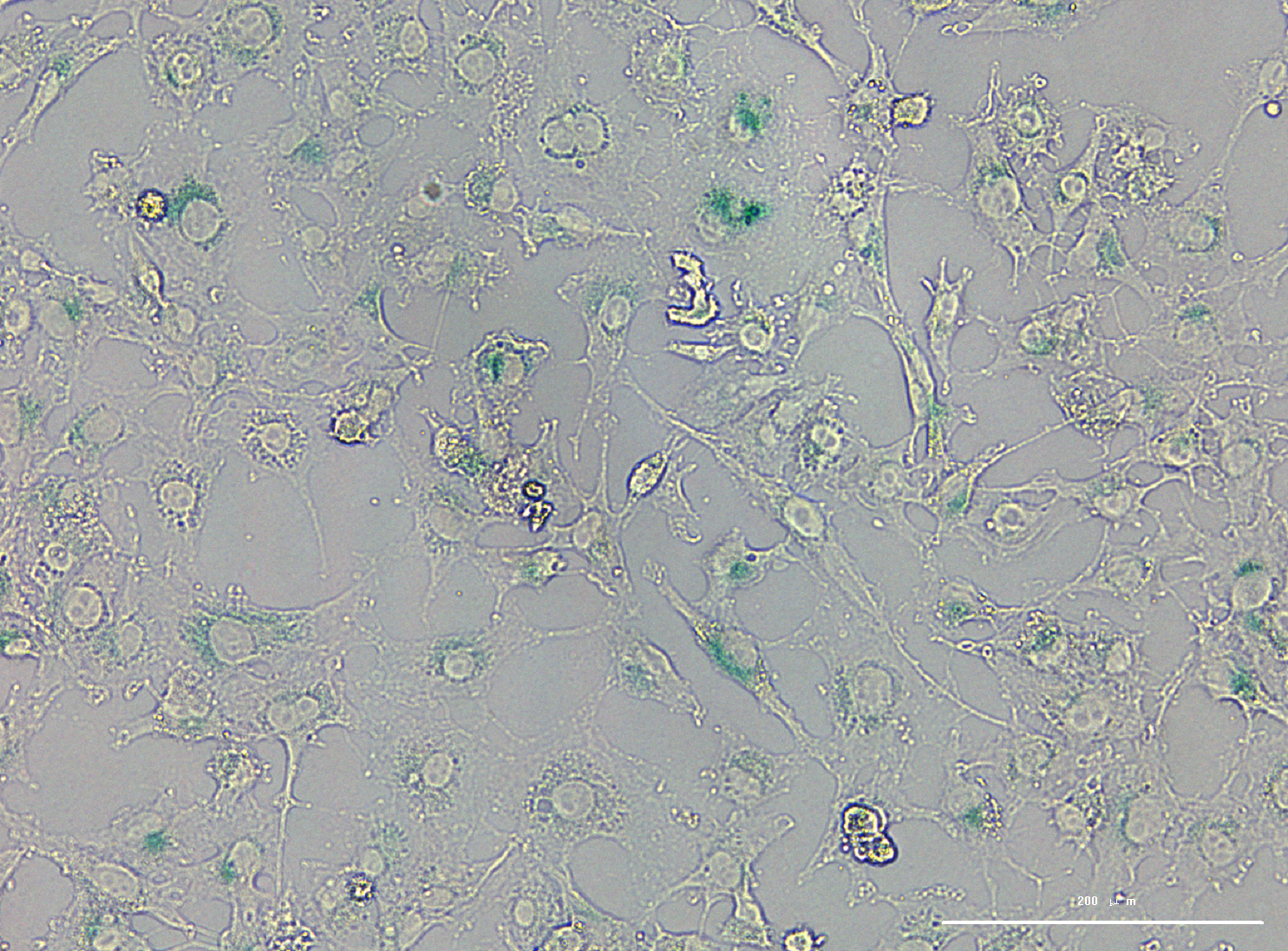

Supplement: Supplementary file 47 — Supplementary file47 (PNG 4526 KB) [file 11033_2025_10810_MOESM47_ESM.png]

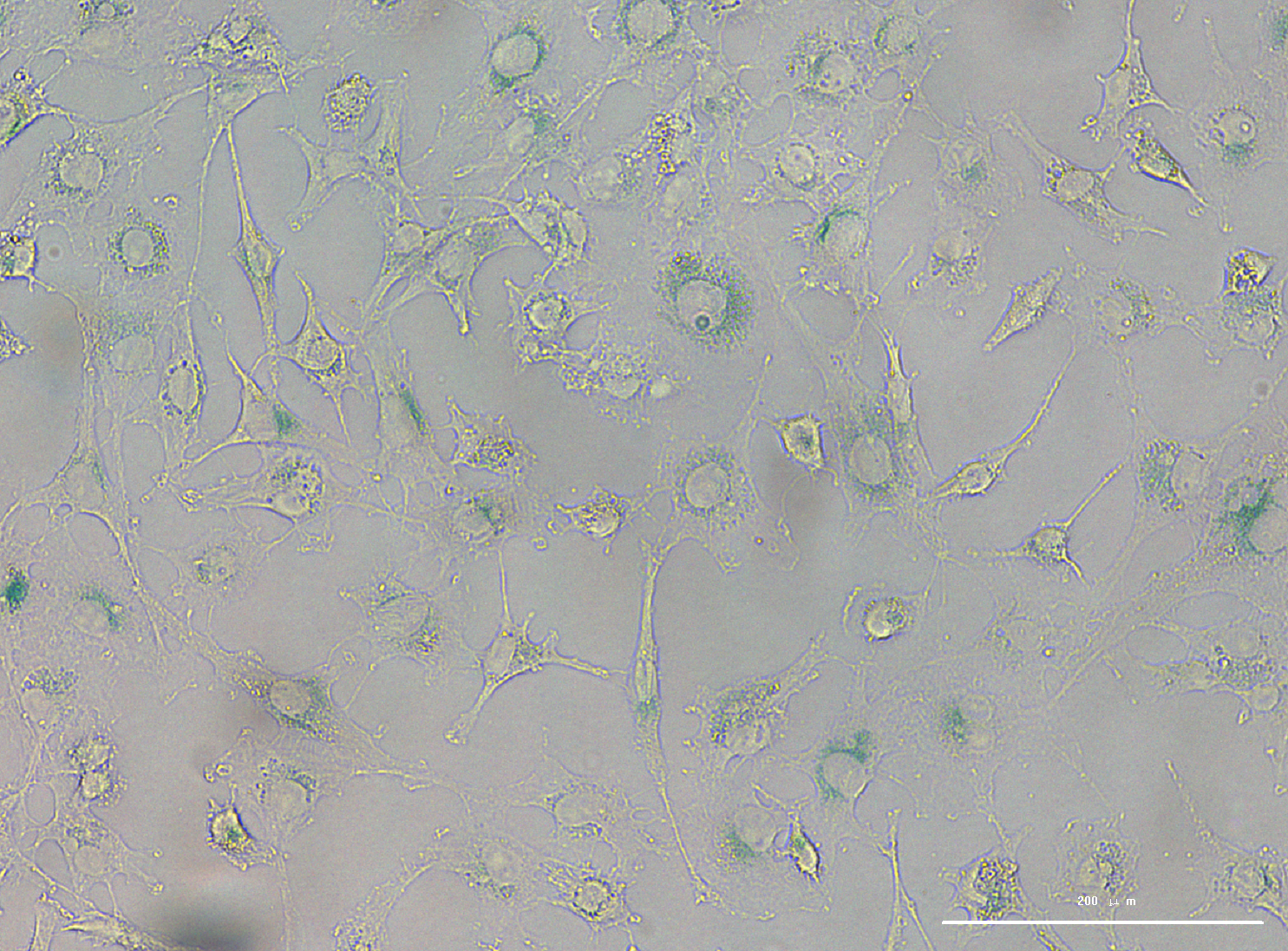

Supplement: Supplementary file 48 — Supplementary file48 (PNG 4393 KB) [file 11033_2025_10810_MOESM48_ESM.png]

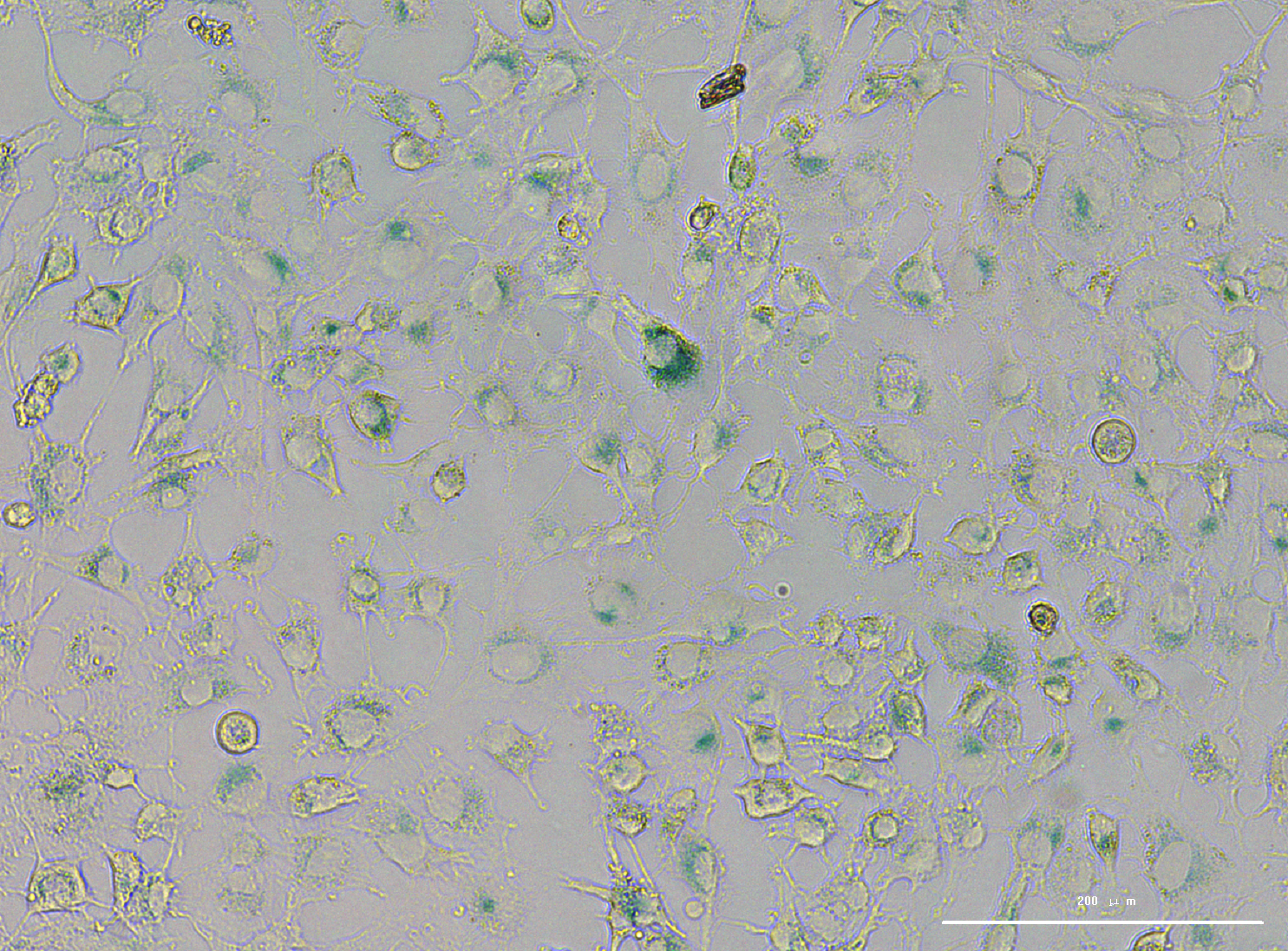

Supplement: Supplementary file 49 — Supplementary file49 (PNG 4643 KB) [file 11033_2025_10810_MOESM49_ESM.png]

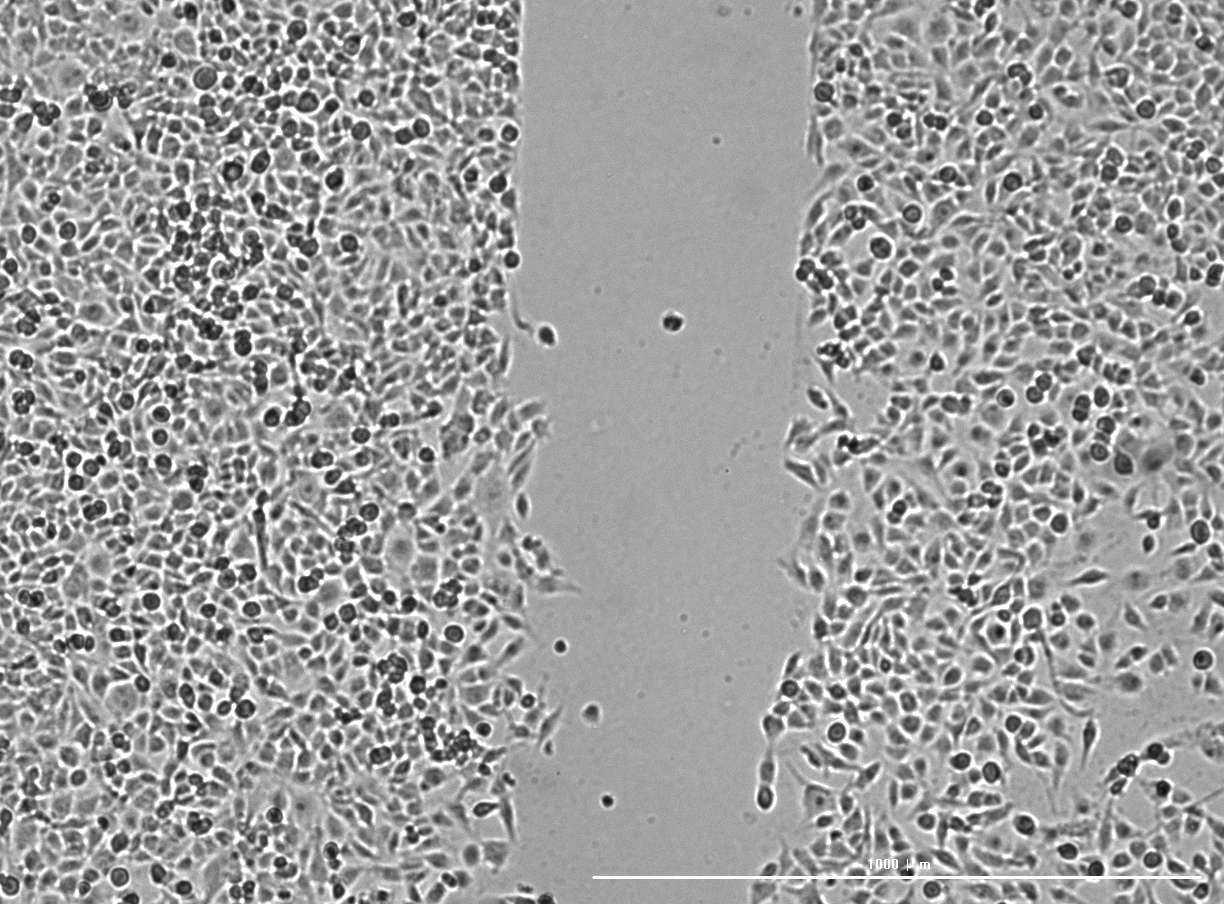

Supplement: Supplementary file 50 — Supplementary file50 (PNG 1452 KB) [file 11033_2025_10810_MOESM50_ESM.png]

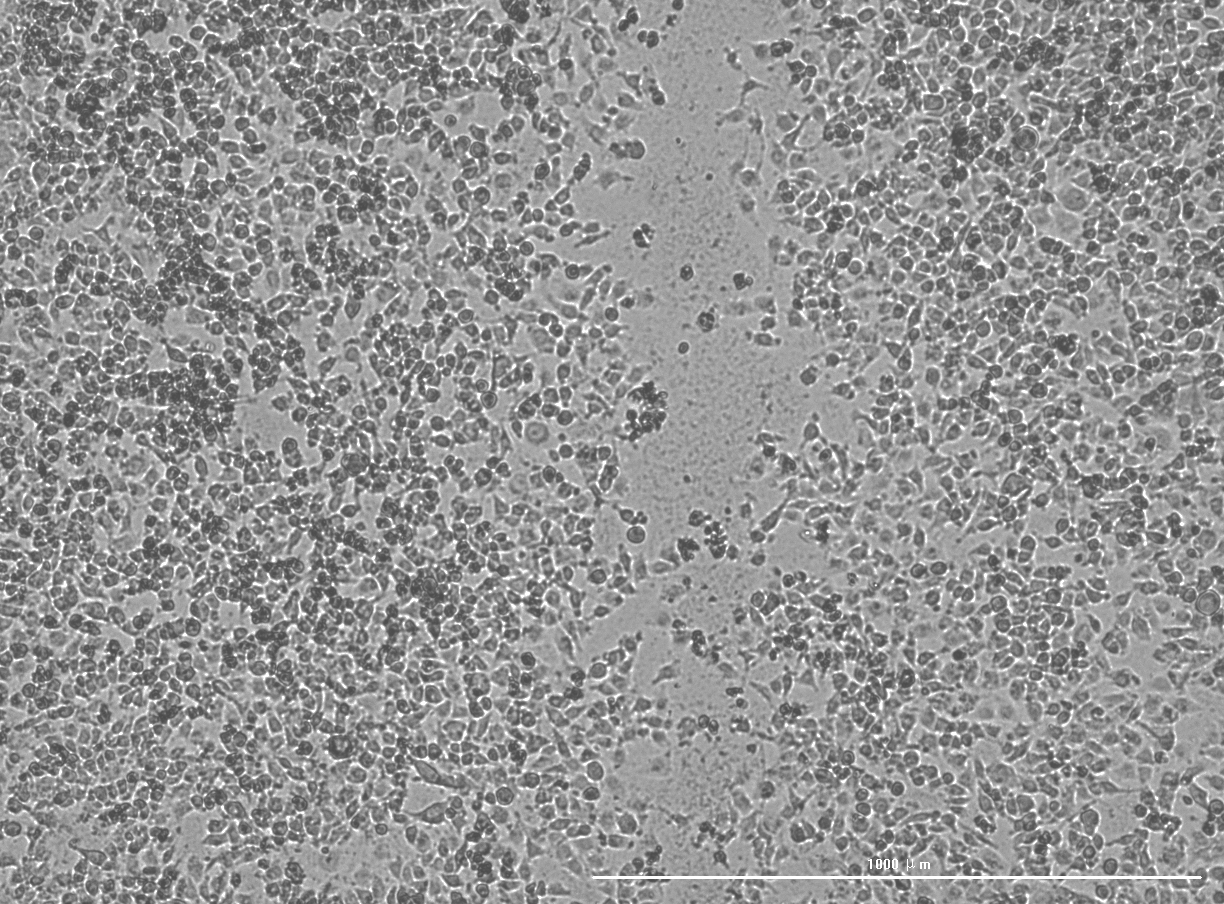

Supplement: Supplementary file 51 — Supplementary file51 (PNG 1618 KB) [file 11033_2025_10810_MOESM51_ESM.png]

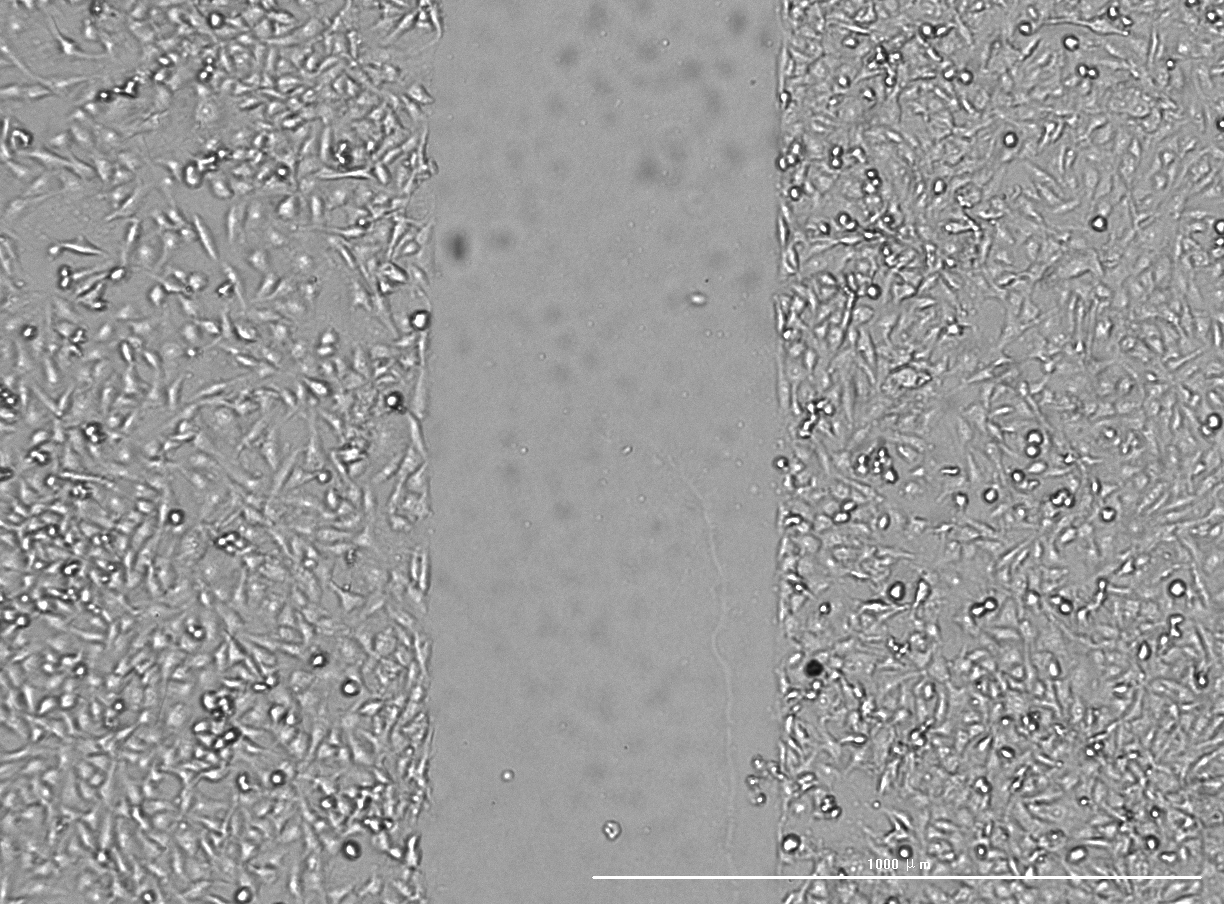

Supplement: Supplementary file 52 — Supplementary file52 (PNG 1377 KB) [file 11033_2025_10810_MOESM52_ESM.png]

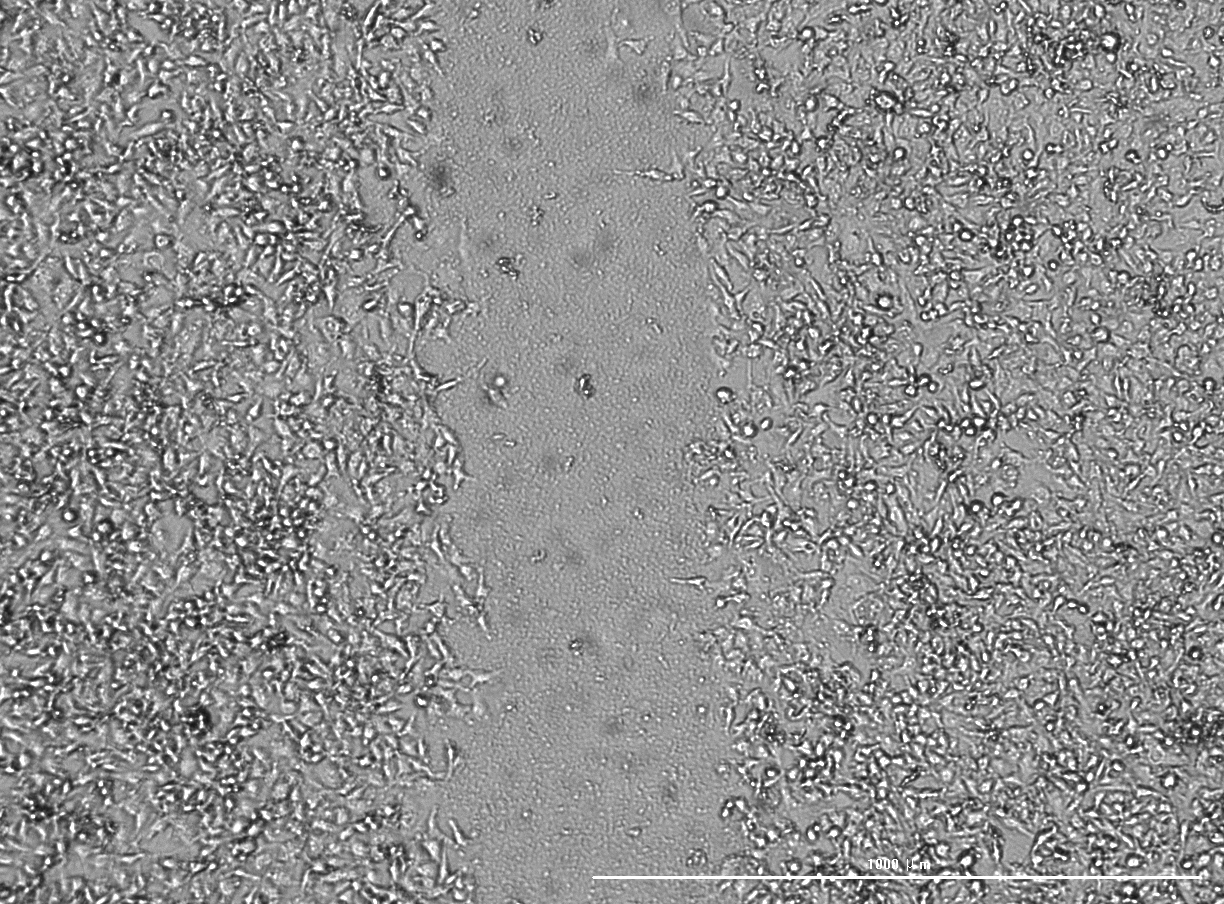

Supplement: Supplementary file 53 — Supplementary file53 (PNG 1519 KB) [file 11033_2025_10810_MOESM53_ESM.png]

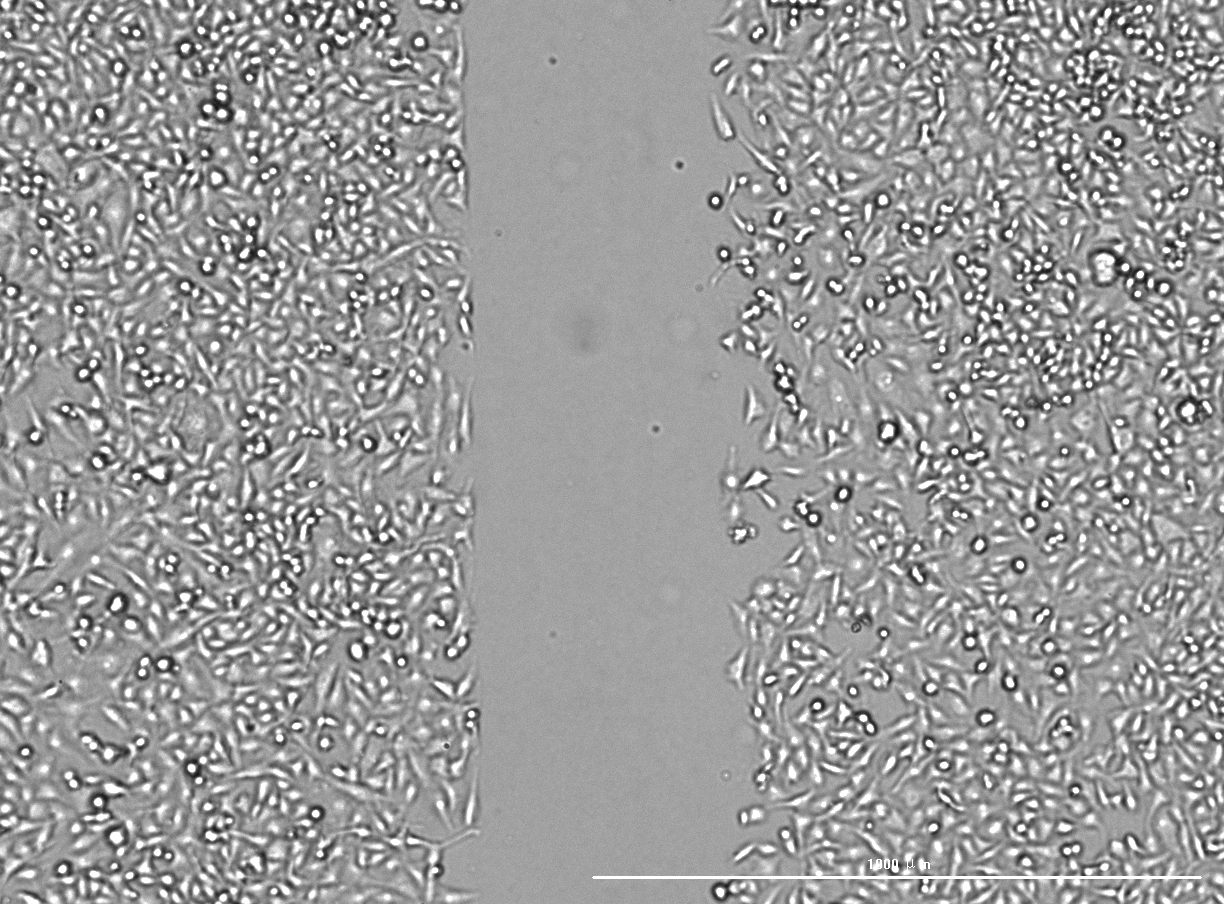

Supplement: Supplementary file 54 — Supplementary file54 (PNG 1410 KB) [file 11033_2025_10810_MOESM54_ESM.png]

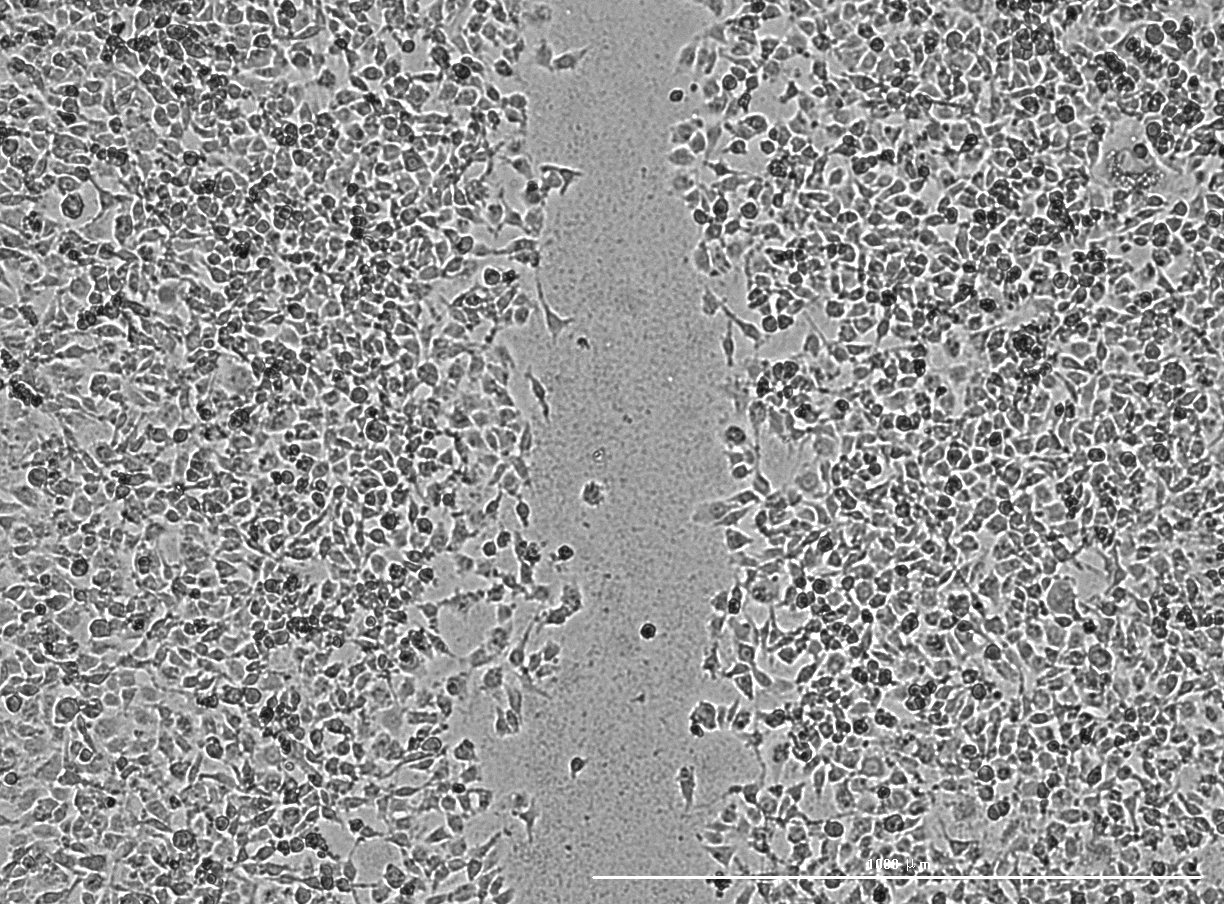

Supplement: Supplementary file 55 — Supplementary file55 (PNG 1527 KB) [file 11033_2025_10810_MOESM55_ESM.png]

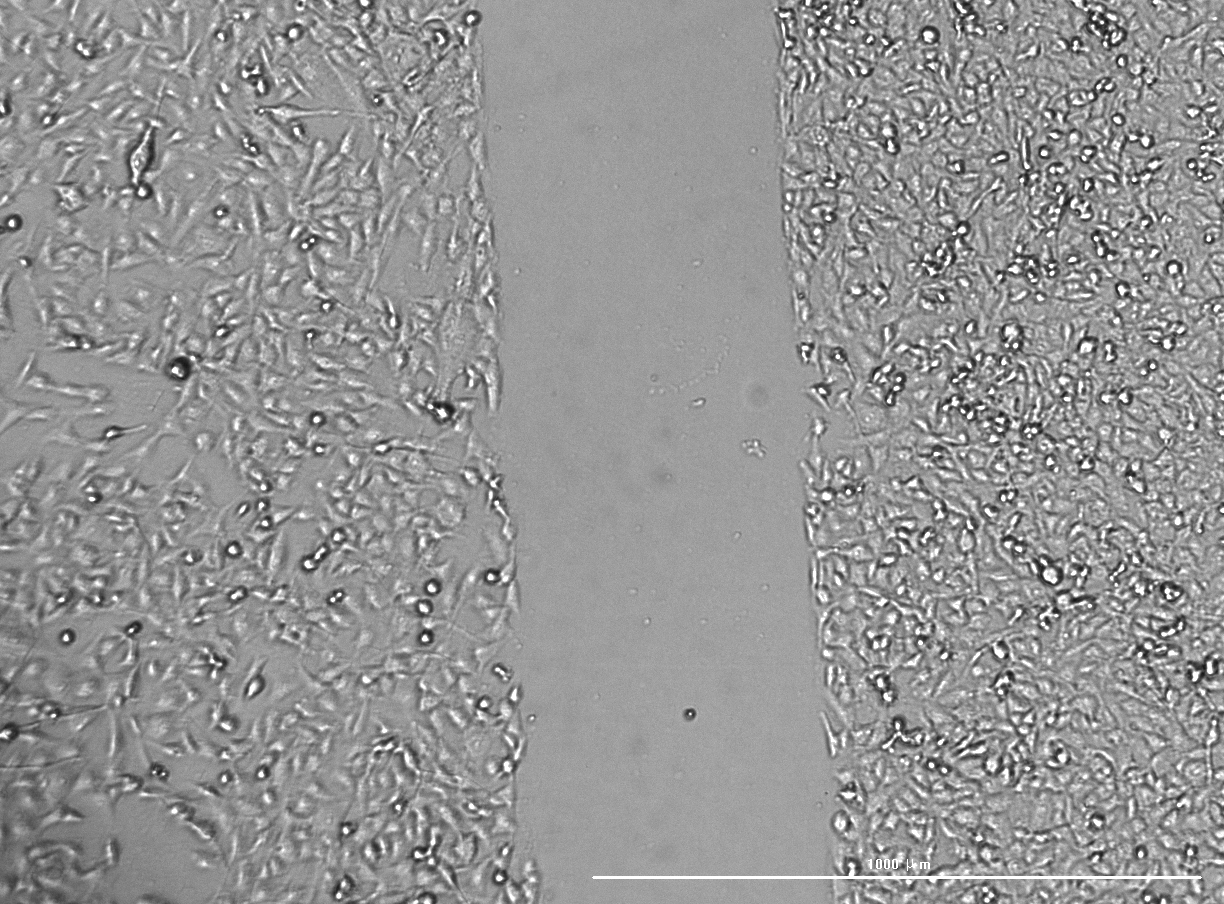

Supplement: Supplementary file 56 — Supplementary file56 (PNG 1396 KB) [file 11033_2025_10810_MOESM56_ESM.png]

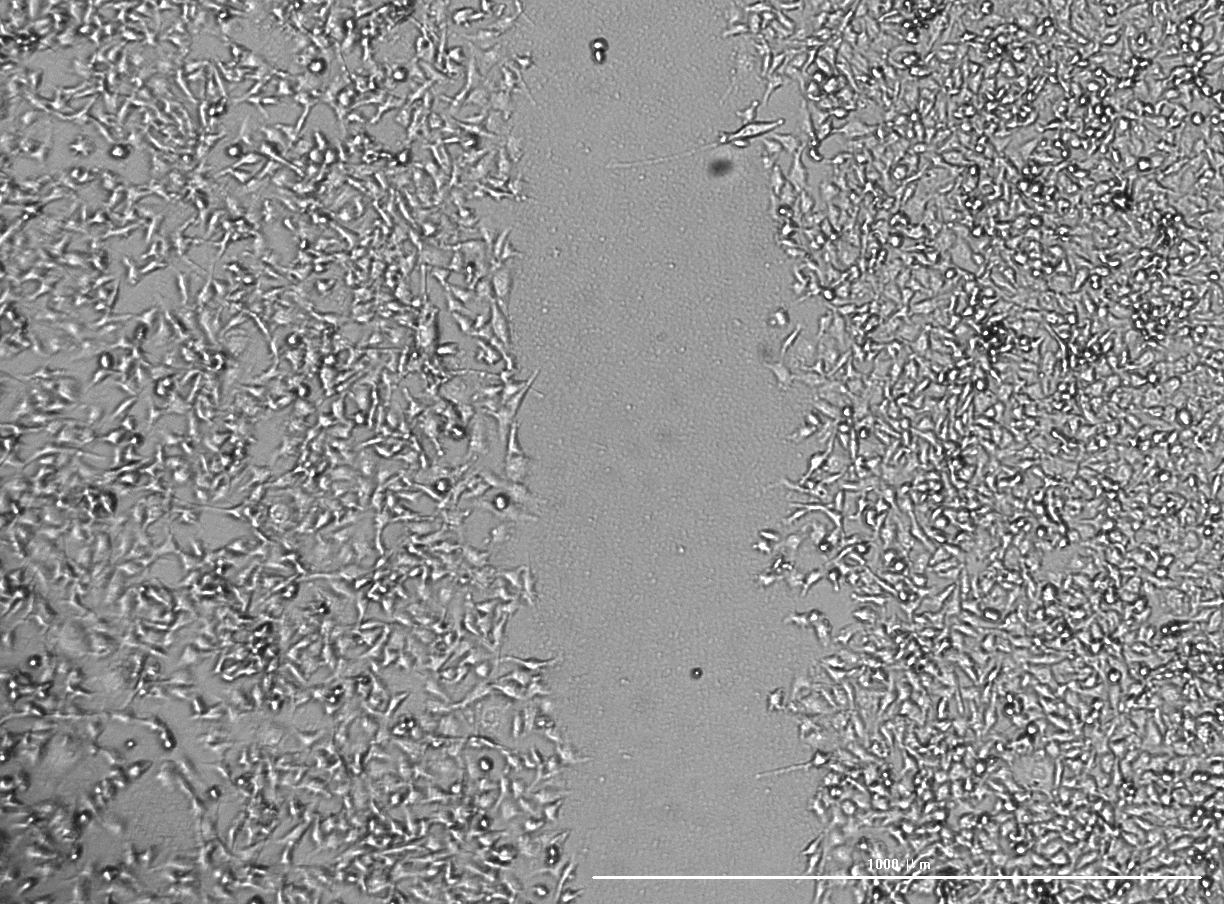

Supplement: Supplementary file 57 — Supplementary file57 (PNG 1476 KB) [file 11033_2025_10810_MOESM57_ESM.png]

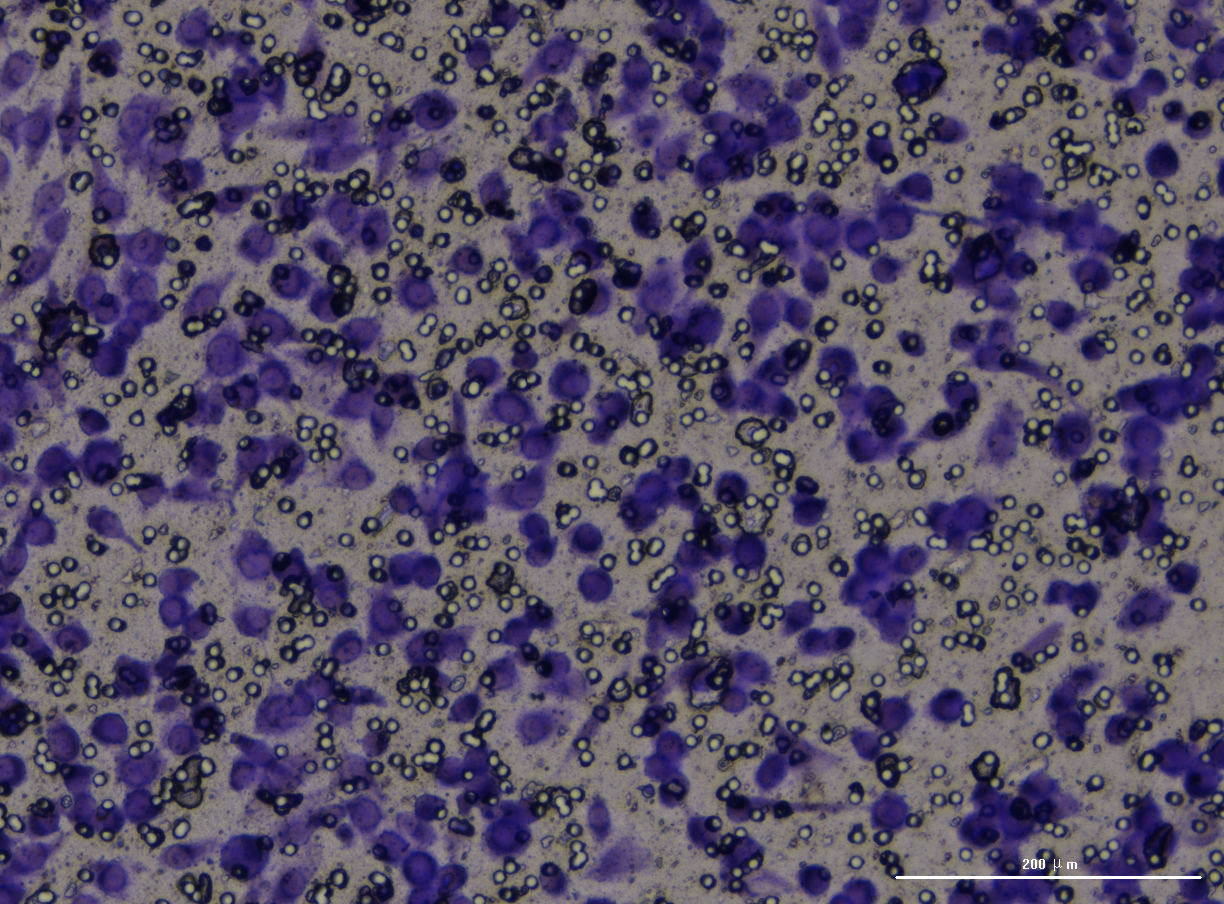

Supplement: Supplementary file 58 — Supplementary file58 (PNG 3163 KB) [file 11033_2025_10810_MOESM58_ESM.png]

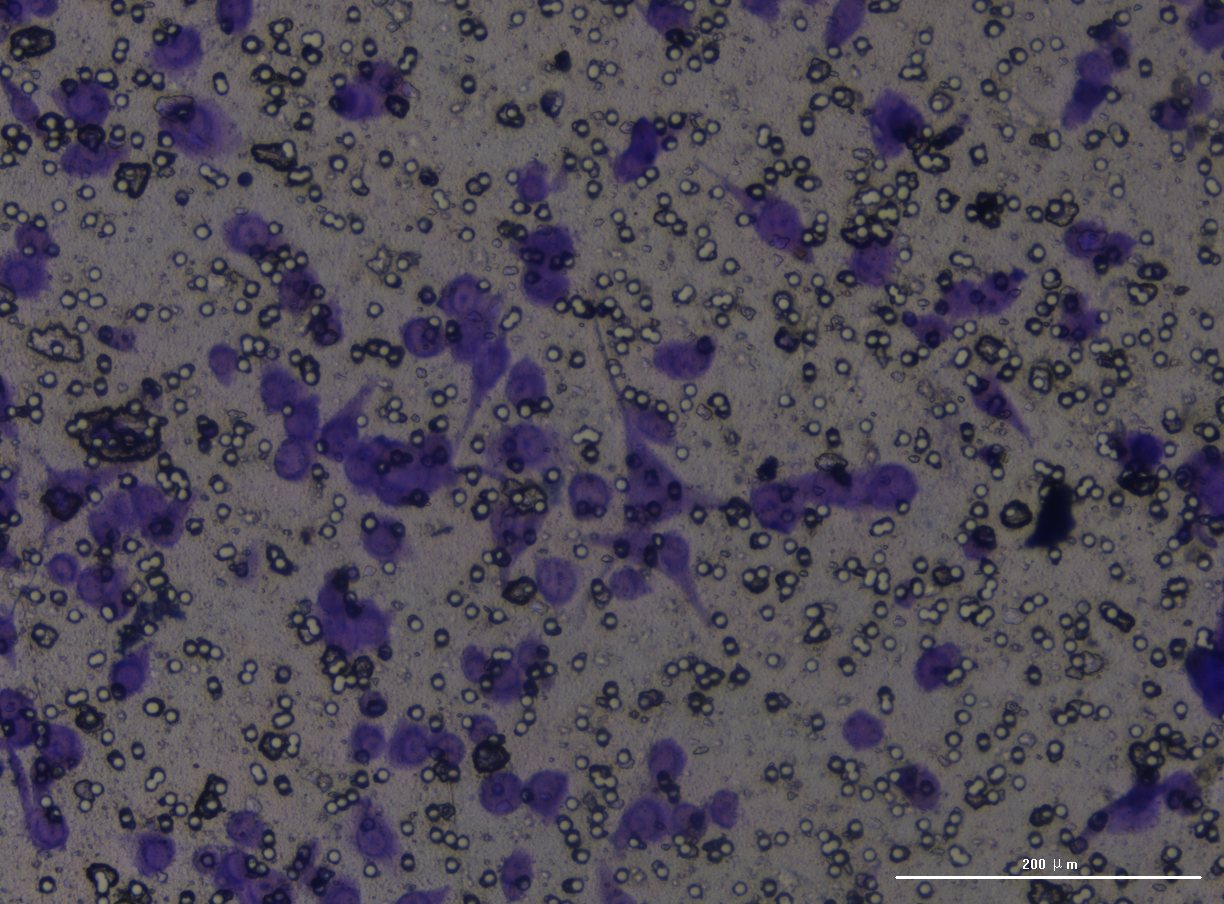

Supplement: Supplementary file 59 — Supplementary file59 (PNG 2888 KB) [file 11033_2025_10810_MOESM59_ESM.png]

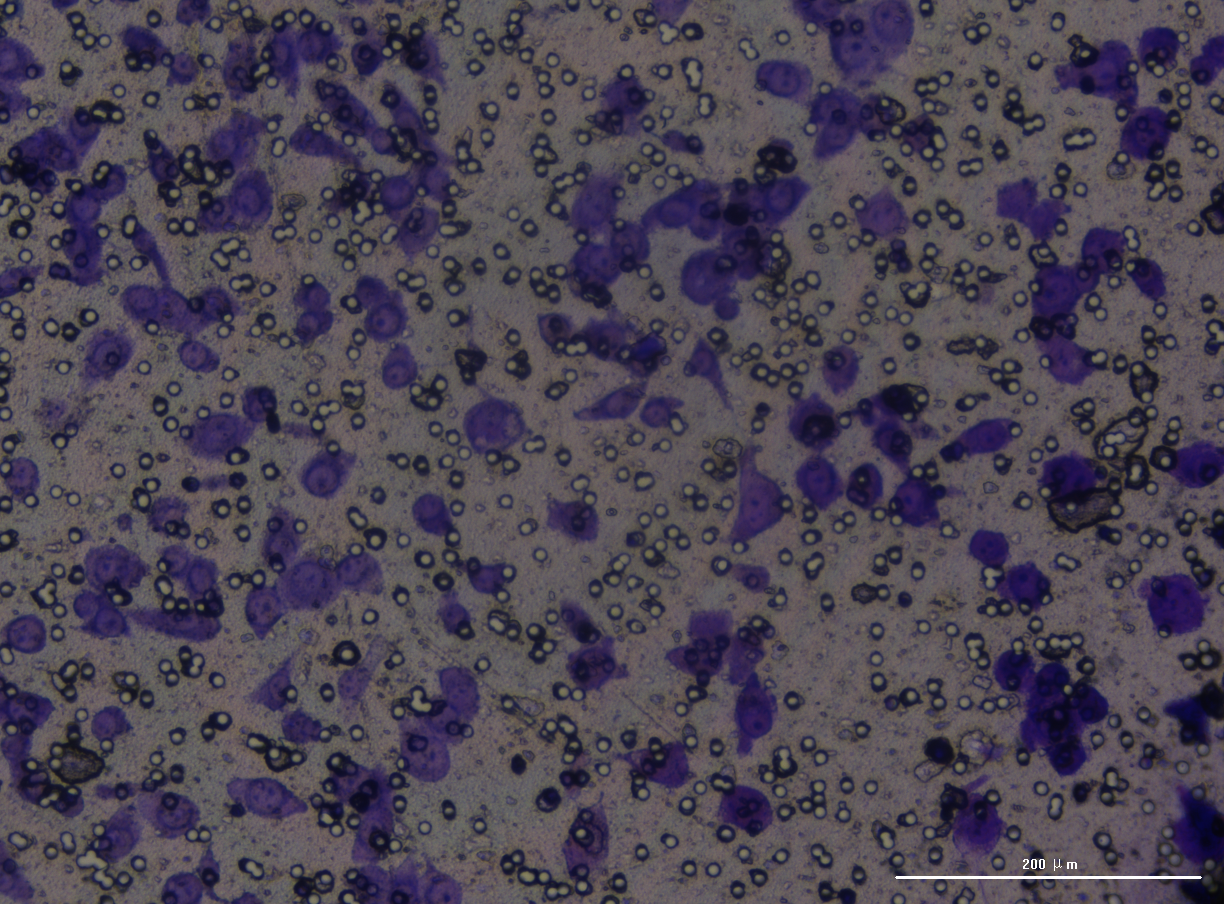

Supplement: Supplementary file 60 — Supplementary file60 (PNG 2924 KB) [file 11033_2025_10810_MOESM60_ESM.png]

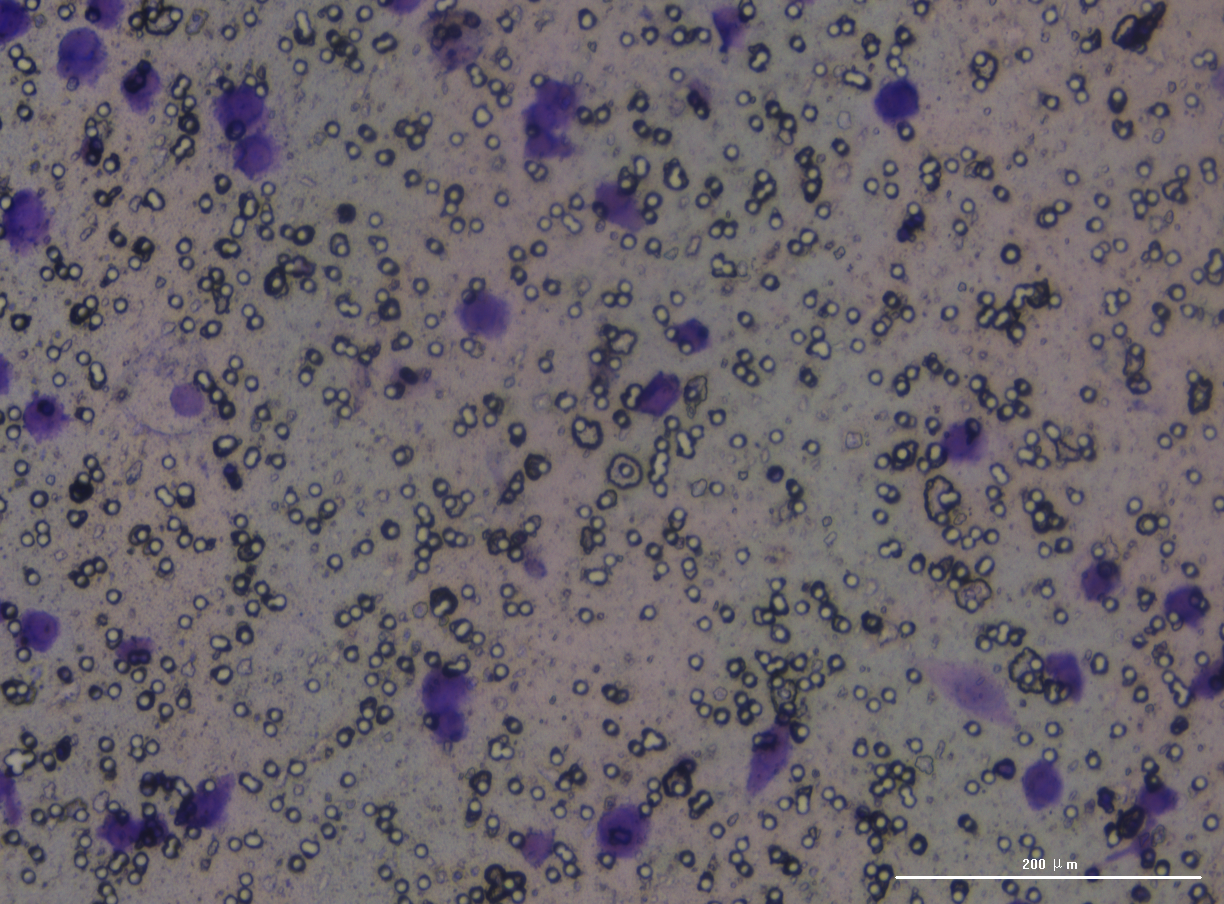

Supplement: Supplementary file 61 — Supplementary file61 (PNG 2882 KB) [file 11033_2025_10810_MOESM61_ESM.png]
